# Bisphenol A impaired cell adhesion by altering the expression of adhesion and cytoskeleton proteins on human podocytes

**DOI:** 10.1038/s41598-020-73636-6

**Published:** 2020-10-06

**Authors:** Rafael Moreno-Gómez-Toledano, María I. Arenas, Clara González-Martínez, Nuria Olea-Herrero, Paula Reventún, Michele Di Nunzio, Sandra Sánchez-Esteban, Eduardo Arilla-Ferreiro, Marta Saura, Ricardo J. Bosch

**Affiliations:** 1 Laboratory of Renal Physiology and Experimental Nephrology, Department of System Biology/Physiology Unit, University of Alcalá, Alcalá de Henares, Spain; 2 Department of Biomedicine and Biotechnology/Cell Biology Unit, University of Alcalá, Alcalá de Henares, Spain; 3 Laboratory of Pathophysiology of the Vascular Wall, Department of System Biology/Physiology Unit, University of Alcalá, Alcalá de Henares, Spain

**Keywords:** Proteins, Proteomics, RNA, Podocytes, Environmental chemistry, Physiology, Cell adhesion

## Abstract

Bisphenol A (BPA), a chemical -xenoestrogen- used in food containers is present in the urine of almost the entire population. Recently, several extensive population studies have proven a significant association between urinary excretion of BPA and albuminuria. The alteration of glomerular podocytes or "podocytopathy" is a common event in chronic albuminuric conditions. Since many podocytes recovered from patients' urine are viable, we hypothesized that BPA could impair podocyte adhesion capabilities. Using an in vitro adhesion assay, we observed that BPA impaired podocyte adhesion, an effect that was abrogated by Tamoxifen (an estrogen receptor blocker). Genomic and proteomic analyses revealed that BPA affected the expression of several podocyte cytoskeleton and adhesion proteins. Western blot and immunocytochemistry confirmed the alteration in the protein expression of tubulin, vimentin, podocin, cofilin-1, vinculin, E-cadherin, nephrin, VCAM-1, tenascin-C, and β-catenin. Moreover, we also found that BPA, while decreased podocyte nitric oxide production, it lead to overproduction of ion superoxide. In conclusion, our data show that BPA induced a novel type of podocytopathy characterizes by an impairment of podocyte adhesion, by altering the expression of adhesion and cytoskeleton proteins. Moreover, BPA diminished production of podocyte nitric oxide and induced the overproduction of oxygen-free metabolites. These data provide a mechanism by which BPA could participate in the pathogenesis and progression of renal diseases.

## Introduction

Podocytes in the kidney glomerulus -also known as glomerular visceral epithelial cells- form the final barrier to protein loss, which explains why a podocyte injury or podocytopathy was accompanied by proteinuria^1,2^. Podocytes are mesenchymal-like differentiated cells and have a unique cellular architecture consisting of a cell body, major and foot processes^1,2^. They also contain the three major components of the eukaryotic cytoskeleton, i.e., intermediate filaments, microtubules, and microfilaments, or actin fibers. About 100 actin-associated proteins have been described in podocytes (i.e., alfa-actinin-4, ezrin, kindlin-2, filamin-D, cofilin-1, vinculin, profilin-1). Podocytes are anchored to specific matrix proteins of the glomerular basement membrane (i.e., VCAM-1, tenascin-C) and cell–cell binding (i.e., vinculin, E-cadherin, nephrin). Alterations in cell–matrix and cell–cell adherence lead to podocyte detachment^3–6^.

Because of podocytes´ inability to proliferate adequately, podocytopenia follows when cells undergo apoptosis, detachment, necrosis, and altered autophagia in response to injury. This leads to progressive glomerular scarring^1–3^. Podocytopathy could be the result of an increasing list of conditions such as genetic, infectious, immune, and toxic aminoglycosides, including diabetes mellitus, with diabetic nephropathy (DN) being the most common cause of end-stage renal disease in developed countries. Interestingly, loss of podocytes into the urine (podocyturia) has been detected in many glomerular diseases^7–10^.

Bisphenol A (BPA) or 2,2, -bis (4-hydroxyphenyl) propane is a molecule used to synthesize polycarbonate plastics and epoxy resins extensively used in the production water and soft drinks bottles, and as the inner coating of cans and other food and drink containers (reviewed in^11^). It is well established that BPA belongs to the increasing list of endocrine disruptor agents (xenoestrogen). As such, BPA has been implicated with several endocrine and metabolic abnormalities, including hepatic and thyroid disorders, obesity, cardiovascular diseases, and increased susceptibility to diabetes^12–16^.

Numerous studies have demonstrated that more than 95% of the population in the USA, Japan, and Spain have detectable urinary levels of BPA^11,17–19^. Even more concerning is the fact that studies conducted in several countries have shown environmental levels of BPA in water, dust, and air^12,20–24^. The environmental exposure is particularly intense among workers in the plastics industry, as well as people exposed to thermal paper^25–27^. BPA is conjugated by the liver, particularly after oral exposure, losing its estrogenic activity, and is then excreted mainly through the intestine. Both non-conjugated (bioactive) and conjugated BPA (inactive) are excreted in the urine^28–30^.

Although several large population studies have demonstrated a significant association between urinary excretion of BPA concentration and albuminuria^31–34^, there are no direct studies on the potential effect of BPA on human podocytes. Since several investigators have reported that many podocytes recovered from patients' urine are viable^35–39^ herein, we hypothesize that BPA could impair podocyte ability to remain attached to the underlined glomerular basement membrane. To test this hypothesis, we performed an in vitro adhesion assay to measure the anchorage of cultured human podocytes to the culture flask. We also studied the potential cellular mechanism by which BPA promotes a novel type of podocytopathy characterized by cell adhesion impairment.

## Results

### BPA impaired podocyte adhesion

**Figure 1:**
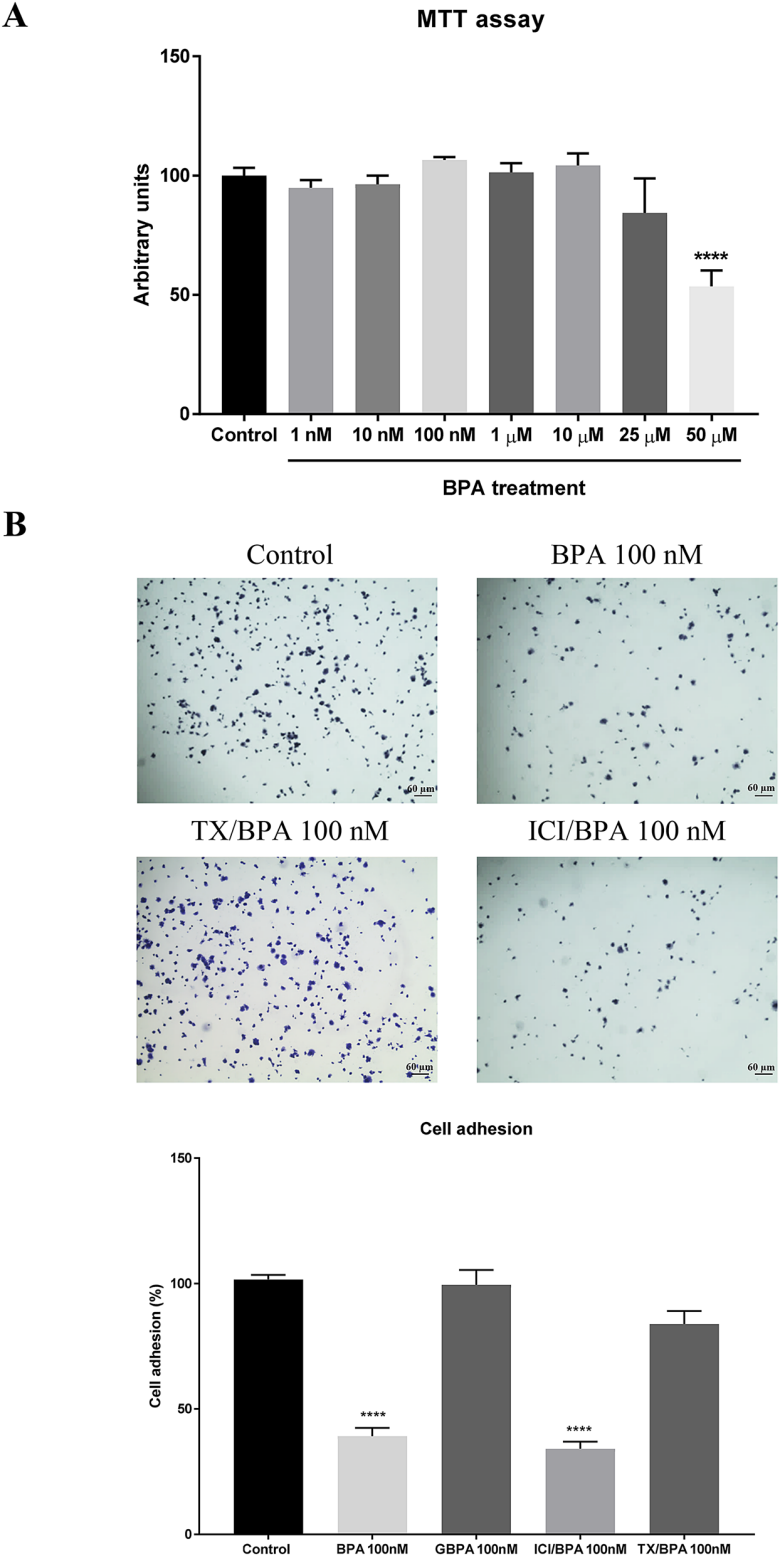
BPA impaired podocyte adhesion. (**A**) MTT assay showing the percentage of cell viability with BPA treatment to different concentrations. (**B**) Adhesion assay: micrographs of podocytes (×40) incubated with BPA as well as pre-treatment 1 h with estrogen receptor blockers Tamoxifen (TX) 100 nM or ICI 100 nM. Note that the result of GBPA, a metabolized form of BPA, has been included in the graph. Data are the means ± SEM of three different experiments, each performed in duplicate. ****p < 0.0001 using ANOVA test.

MTT assays showed a decrease in viability at concentrations of BPA higher than 50 µM. No significant differences were observed with lower concentrations (Fig. 1A).

Cell–cell contacts and the adherence of podocytes to the extracellular matrix of the glomerular basement membrane are crucial for podocyte function^3–6^. Herein, adhesion assays were performed to investigate the effect of low concentration of BPA on human podocyte anchorage to the culture flask. As shown in Fig. 1B, we first observed that a concentration of 100 nM BPA decreased podocyte adhesion by 50%. No significant effects were observed at lower BPA concentration (data not shown).

Because most of the BPA is glucuronidated in the liver, where it loses its estrogenic activity, we wanted to explore if liver detoxification can prevent podocyte injury. For this reason, human podocytes were incubated with 100 nM and 1 µM glucuronide BPA for 48 h. No significant effects on the podocyte adhesion were observed. Because BPA is capable of activating the estrogen receptors (ER), we next wanted to investigate if the observed effect of BPA on podocyte adhesion was due to the estrogenic effect of BPA. To this end, we used Tamoxifen and ICI 182,780 (Fulvestrant), compounds capable of blockade ER^40,41^. We pretreated podocytes with either Tamoxifen 100 nM or ICI 100 nM for 1 h before cells were treated with BPA 100 nM for 48 h. We observed that Tamoxifen, but not ICI, was able to abrogate the effect of BPA on podocyte adhesion.

### Transcriptomic and proteomic effects of BPA on human podocytes

To identify the mechanism by which BPA impaired podocyte adhesion, we performed a transcriptomic and a proteomic analysis on human podocytes.

The transcriptomic analysis of human podocytes treated with BPA revealed a decrease in the transcription of genes that codified for proteins that interact and stabilize nephrin (involved in podocyte-podocyte binding). Moreover, the Gene Set Enrichment Analysis (GSEA) also revealed an increase in the transcription of genes that encode for proteins involved in the formation of tubulin folding intermediates (Table 1). Other proteins involved in cell adhesion, such as annexin-A2, filamin-C, as well as galectin-1, were also affected (data not shown).

**Table 1:** Transcriptomic analysis of cell–cell adhesion and cytoskeleton clusters. For primary data processing, image analysis, per-cycle base calling, and quality score assignment was performed with Illumina Real-Time Analysis software (https://emea.illumina.com/).

GS	SIZE	ES	NES	NOM *p* val	FDR *q* val	FWER *p* val	RANK AT MAX	LEADING EDGE
Reactome Nephrin interactions	19	− 0.38	− 2.03	0.004	0.030	0.661	10,941	tags = 74%list = 35% signal = 114%
Reactome formation of tubulin folding intermediates	19	0.40	2.07	0.004	0.011	0.571	7222	tags = 63%list = 23% signal = 82%

Due to the growing list of molecules involves in the mechanism of cell adhesion, we performed a proteomic analysis of human podocytes incubated with BPA. Overall, the proteomic analysis showed significant changes in the expression of 75 proteins related to lipid and glucose metabolism, calcium-binding, cytoskeleton, and adhesion. Only the corresponding proteins with a q-value < 0.05 were considered when contemplating p-value < 0.05. The list of statistically significant proteins (with a higher False Discovery Rate) reached 363 proteins (data not shown).

Table 2 shows a list of 27 proteins -related to cell adhesion function- in which BPA significantly altered the pattern of expression, of which 21 of them proved to be downregulated while the remaining six were upregulated. Although the transcriptomic analysis showed less sensitivity in-display genomic changes induced by BPA compared to the proteomic studies, we found interesting functional coincidences in the altered pattern expression of proteins related to cell adhesion and the cytoskeleton.

**Table 2:** Proteomic analysis of cell adhesion, cytoskeleton and oxidative stress proteins.

Protein	Fold-change	*p* value	*q* value
Nesprin	− 0.958	0.00004	0.004
FRAS1-related extracellular matrix protein 3	− 0.736	0.000709993	0.026
Transgelin	− 0.519	0.00001	0.002
Collagen alpha-1(I) chain	− 0.385	0.00004	0.004
Tropomyosin beta chain	− 0.298	0.001379986	0.041
Cell surface glycoprotein MUC18	− 0.251	0.000419996	0.02
Cofilin-1	− 0.241	0.000559994	0.024
Annexin A1	− 0.223	0.00002	0.002
Elongation factor 2	− 0.217	0.00001	0.001
Vimentin	− 0.215	0.00001	0.004
Profilin-1	− 0.199	0.000199998	0.011
Galectin-1	− 0.196	0.000549995	0.024
Ezrin	− 0.185	0.00097999	0.032
Annexin 2	− 0.183	0.00001	0.001
Elongation factor 1-gamma	− 0.174	0.001779982	0.048
T-complex protein 1 subunit gamma	− 0.173	0.000679993	0.026
L-lactate dehydrogenase A chain	− 0.17	0.000889991	0.03
Glyceraldehyde-3-phosphate dehydrogenase	− 0.159	0.000859991	0.03
AHNAK	− 0.152	0.00001	0.002
Vinculin	− 0.144	0.000149999	0.009
Filamin-C	− 0.134	0.000489995	0.023
Microtubule-associated protein 4	0.154	0.000499995	0.023
Transforming growth factor-beta-induced protein ig-h3	0.193	0.000659993	0.025
Glutathione synthetase	0.197	0.00064999	0.025
Src substrate cortactin	0.206	0.00009	0.007
Fibronectin	0.302	0.00001	0.005
Lactadherin	0.307	0.00009	0.007
Tenascin-C	0.314	0.00001	0.002
NADPH:adrenodoxin oxidoreductase, mitochondrial	0.451	0.00001	0.003
Superoxide dismutase	0.546	0.00001	0.001

Moreover, an increase in the superoxide dismutase, NADPH adrenodoxin oxidoreductase, and glutathione synthetase expression was also observed, which suggest an oxidative stress response. Herein, the potential effect of BPA on SO and NO production on human podocytes was also studied.

### BPA affected the expression of cell–cell adhesion and cytoskeleton proteins as assessed by Western blot and immunocytochemistry

Based upon our main findings obtained in both transcriptomic and proteomic analysis, we proceeded to fully confirm significant changes in protein expression by Western blot as well as immunohistochemistry using the most specific commercially available antibodies.

We found that BPA induced changes in the expression of several critical functional podocyte proteins capable of regulating podocyte cytoskeleton, cytoskeleton stabilization, cell–cell adhesion, and cell–matrix adhesion.

**Figure 2:**
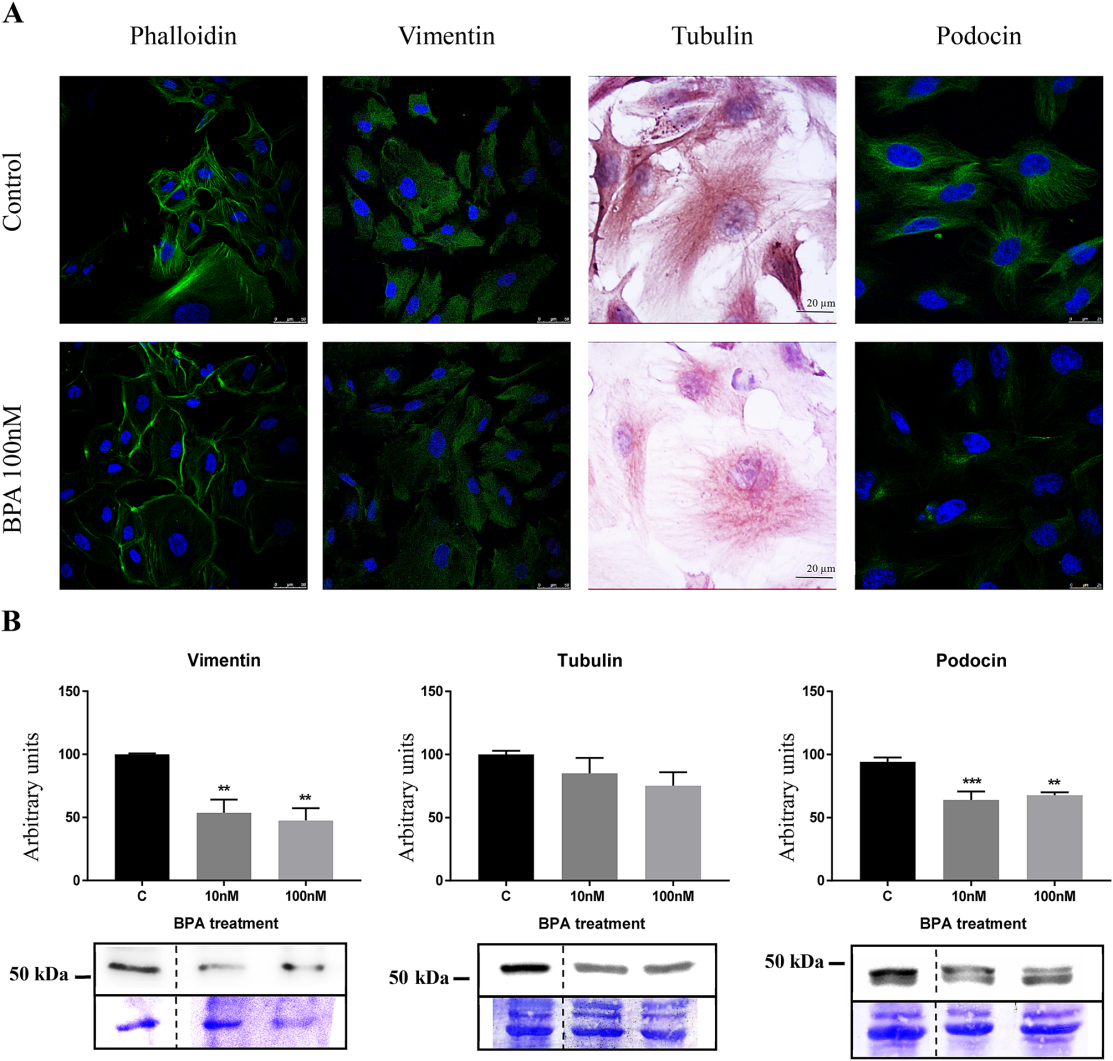
BPA downregulated proteins of podocyte cytoskeleton. (**A**) Immunocytochemistry assay of F-actin (phalloidin), vimentin, tubulin and podocin. (**B**) Western blot of vimentin, tubulin and podocin. Data are the means ± SEM of three different experiments, each performed in duplicate. **p < 0.01 and *** *p* < 0.001 using ANOVA test for the comparison between control and BPA-treated cells. Due to BPA modified the housekeeping proteins (actin, tubulin, etc.) we used coomassie-blue staining to normalized total protein amount. Discontinue line indicates the absent lane of 1 nM BPA treatment. Full-length blots/gels are presented in Supplementary Figs. 1–3.

First, we observed that on podocytes BPA promoted a downregulation in the expression of proteins of the cytoskeleton like F-actin, vimentin, tubulin and podocin (Fig. 2). We observed a significant reduction in the number of F-actin filaments (stained with phalloidin), which were mainly located in the borders of the cells; however, vimentin was concentrated around the nuclei; tubulin staining showed a significant decreased of the tubulin levels in the podocytes after the BPA treatment. Interestingly, podocin, a critical functional protein of fully differentiated podocyte, also displayed a decreased staining on BPA treated cells.

**Figure 3:**
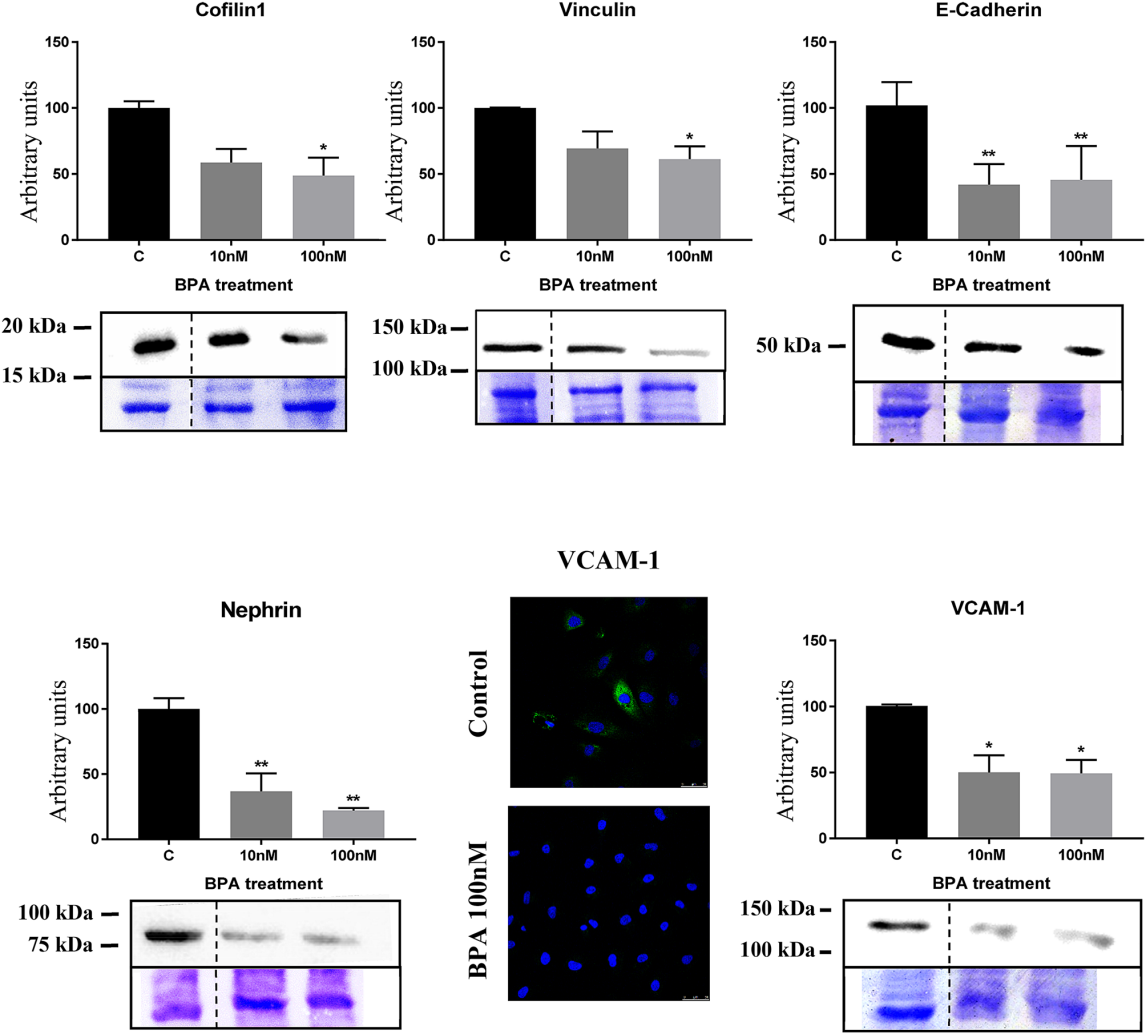
BPA treatment originated a decrease in the protein expression of cofilin-1, vinculin, E-cadherin, nephrin and VCAM-1. Data are the means ± SEM of three different experiments, each performed in duplicate. *p < 0.05 and ** *p* < 0.01 using ANOVA test for the comparison between control and BPA-treated cells. Due to BPA modified the housekeeping proteins (actin, tubulin, etc.) we used coomassie-blue staining to normalized total protein amount. Discontinue line indicates the absent lane of 1 nM BPA treatment. Full-length blots/gels are presented in Supplementary Fig. 4–8.

Second, BPA also induced a significant decreased in the cytoskeleton-binding (stabilization) proteins cofilin-1 and vinculin (Fig. 3).

Third, we found that BPA induced the downregulation of key proteins related to cell adhesion such as E-cadherin, nephrin, and VCAM-1 (Fig. 3).

**Figure 4:**
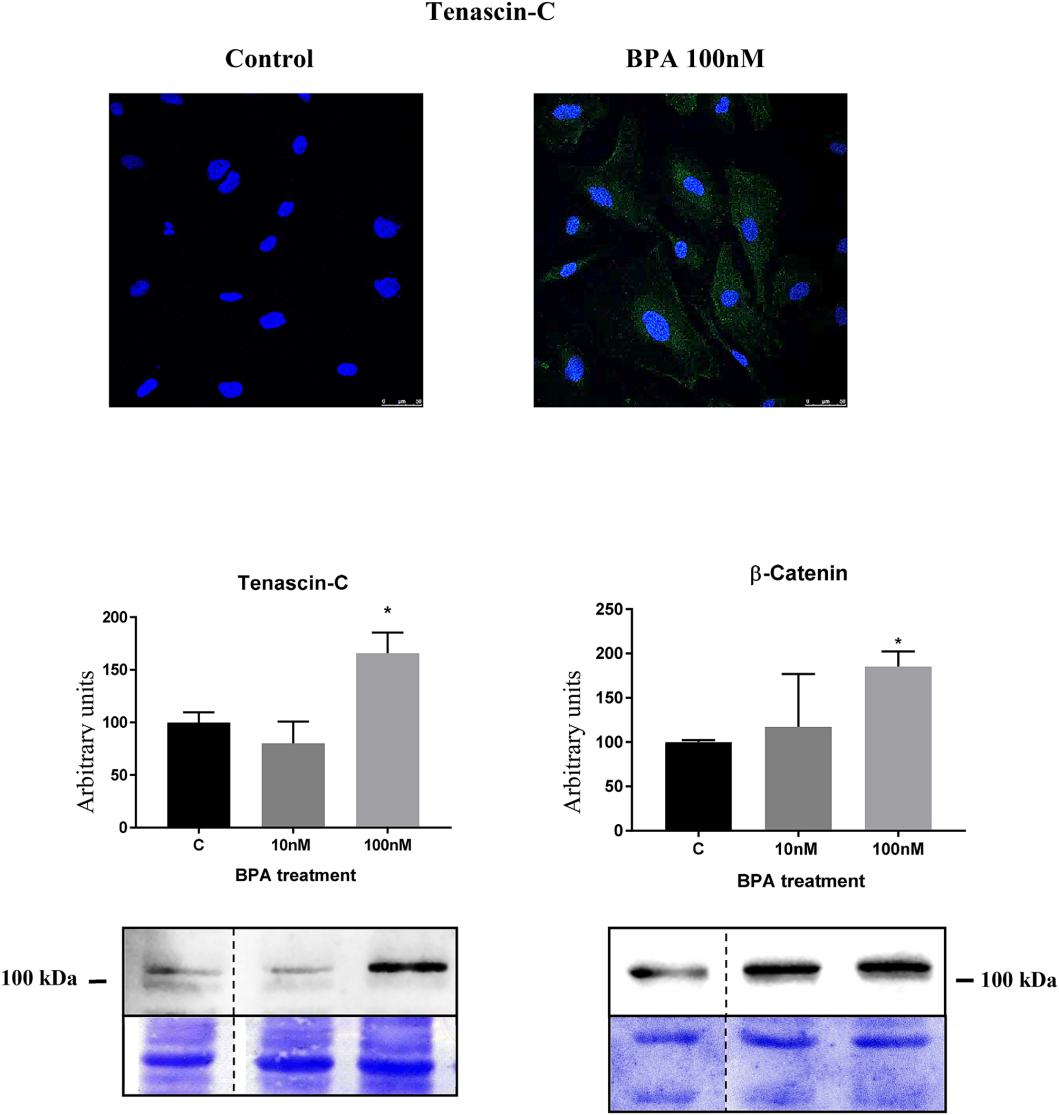
BPA treatment originated an increase in the protein expression of tenascin-C and β-catenin. Data are the means ± SEM of three different experiments, each performed in duplicate. *p < 0.05 using ANOVA test. Due to BPA modified the housekeeping proteins (actin, tubulin, etc.) we used coomassie-blue staining to normalized total protein amount. Discontinue line indicates the absent lane of 1 nM BPA treatment. Full-length blots/gels are presented in Supplementary Figs. 9–10.

Fourth, we found that BPA induced the upregulation of two proteins known to be associated with loss of cell adhesion such as tenascin-C and β-catenin (Fig. 4).

**Figure 5:**
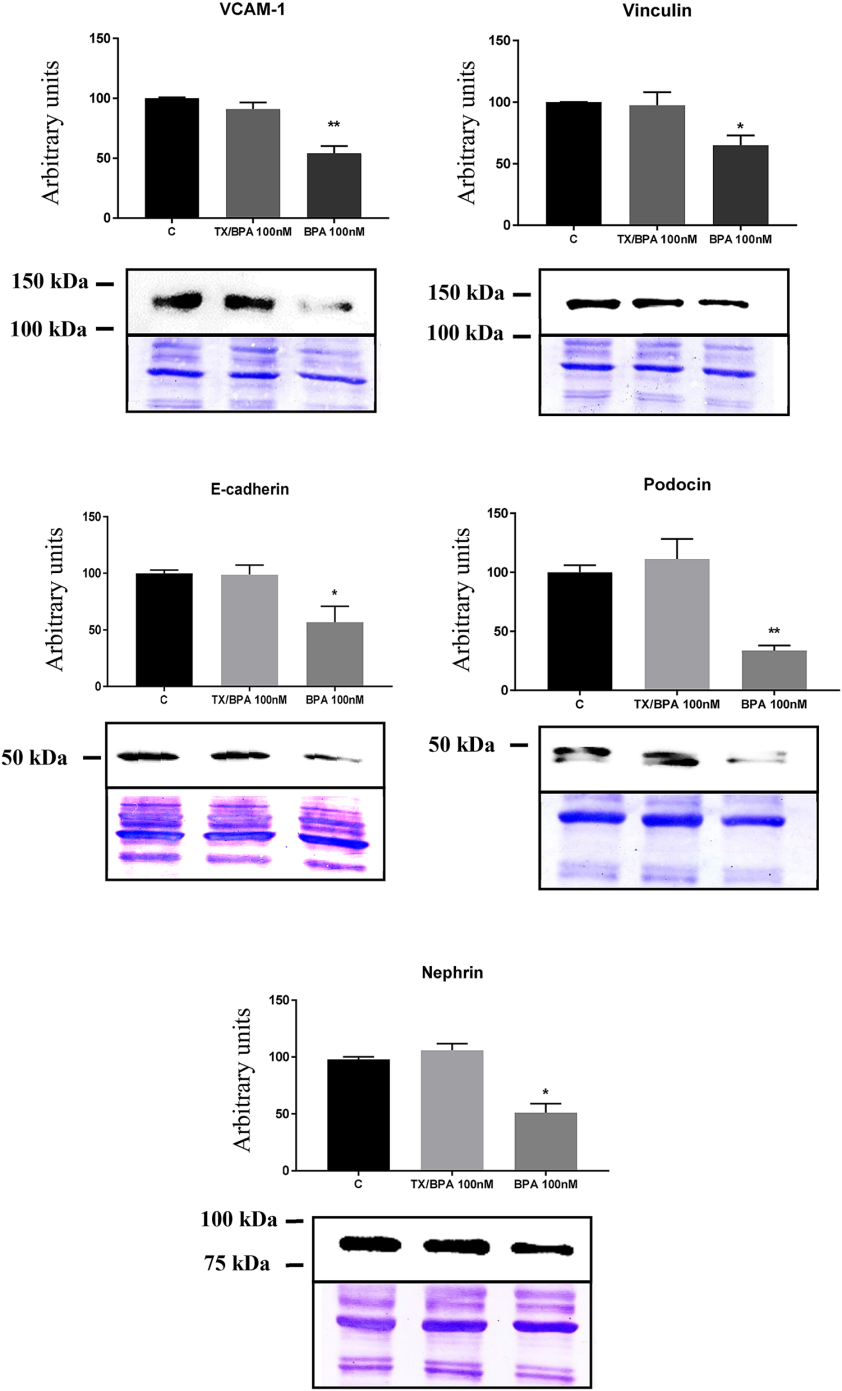
Pre-treatment with Tamoxifen (Tx) inhibited the effect of BPA on adhesion proteins. Data are the means ± SEM of three different experiments, each performed in duplicate. *p < 0.05 and ** *p* < 0.01 using ANOVA test. Due to BPA modified the housekeeping proteins (actin, tubulin, etc.) we used coomassie-blue staining to normalized total protein amount. Full-length blots/gels are presented in Supplementary Figs. 11–15.

Finally, we observed that Tamoxifen was able to abrogate the changes in protein expression (analyzed by Western blot) of VCAM-1, vinculin, E-cadherin, nephrin and podocin induced by BPA on human podocytes (Fig. 5).

### BPA affected NO and SO production on human podocytes

**Figure 6:**
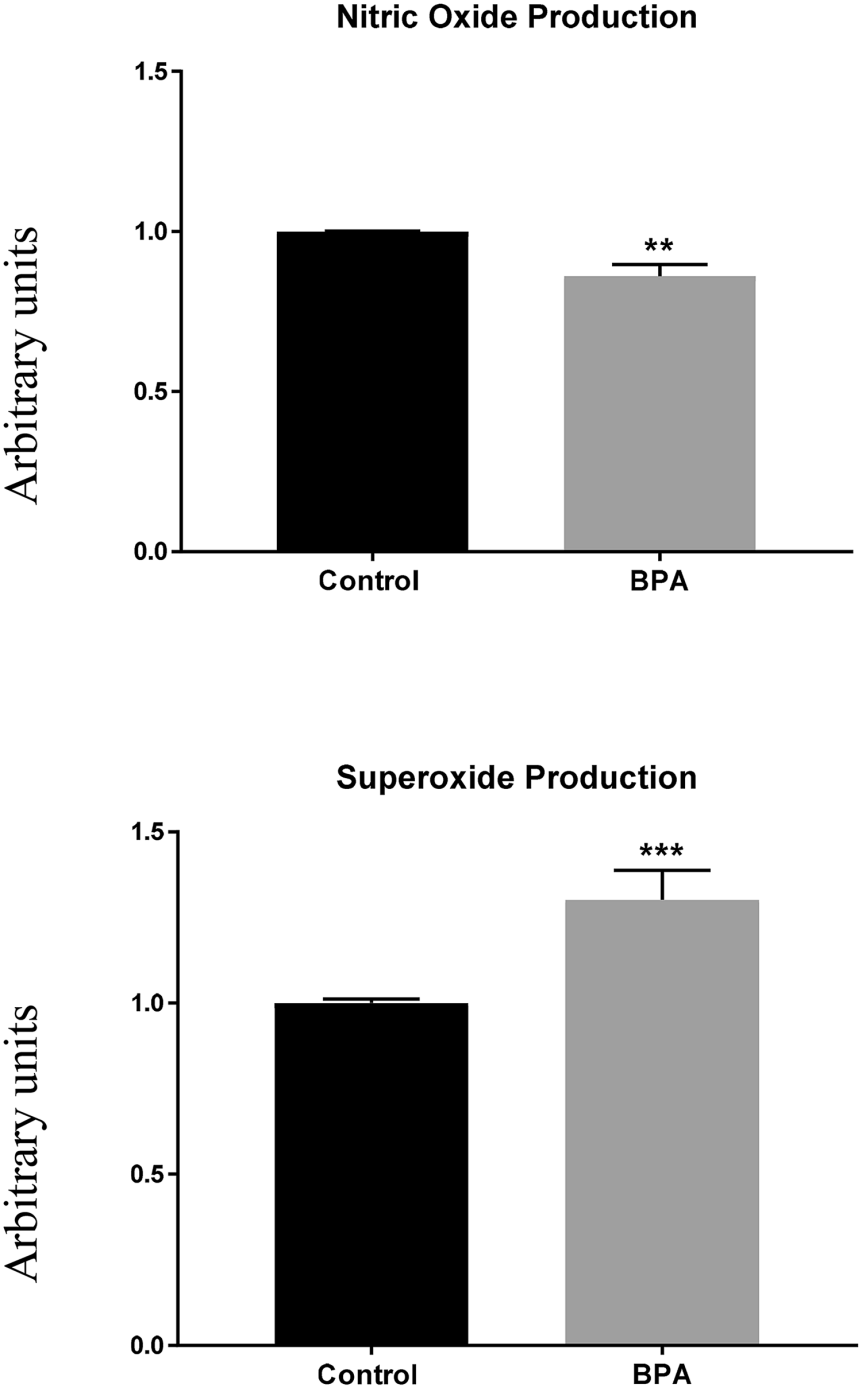
BPA reduced NO production and promoted an increase in the SO concentration. Data are the means ± SEM of three different experiments, each performed in duplicate. ** *p* < 0.01 and *** *p* < 0.001 using ANOVA test.

We measured NO production to explore if BPA activated vasoregulatory genes in human podocytes, observing that BPA decreased its production (Fig. 6). We then measured the output of different free radicals after the BPA exposition by flow cytometry finding that the SO production was significantly increased (Fig. 6).

## Discussion

Experimental animal data have shown that BPA could promote podocyte apoptosis and proteinuria^43^. In humans, several extensive population studies have demonstrated a significant association between urinary excretion of BPA and albuminuria, a well-known factor involved in the mechanism of renal disease progression^31–34^. In this regard, Hu et al.^33^, in a prospective study of 302 patients followed for 6 years, have demonstrated serum BPA as a predictor of chronic kidney disease in primary hypertension. Serum BPA was also described as a risk factor in the progression of diabetic nephropathy in patients with type 2 diabetes^32^. Our present study provides a cellular mechanism of these findings since we describe a novel type of podocytopathy characterized by an impairment of podocyte adhesion capabilities because of a direct BPA alteration on the expression of crucial podocyte functional proteins.

We first performed a reliable and reproducible in vitro adhesion assay to measure the anchorage of cultured human podocytes to the culture flask.

We found that 100 nM BPA significantly impaired podocyte adhesion by 50%. Although no effect was observed at lower BPA concentration, this finding is relevant because such concentration can usually be found in people whose jobs have high exposure to BPA, such as the plastics or thermal papers industry^25–27^. Furthermore, it has been shown that higher concentrations can be found in patients with kidney disease in advanced stages undergoing hemodialysis^44,45^, as well as in neonates undergoing surgical interventions where BPA levels can be even higher^46^.

It is worth noting that the ER antagonist Tamoxifen could abrogate the effect of BPA on podocyte adhesion. Interestingly, glucuronidated BPA (without estrogenic activity) showed no effect on cell adhesion. These findings agree with previous studies showing the adverse effect of BPA through the activation of the ER in other cell types^40,41^.

To investigate the mechanism of BPA´s observed detrimental effect of BPA on human podocyte adhesion, we performed both transcriptomic and proteomic analysis followed by a further verification by Western blot or immunocytochemistry. We found that BPA was able to change significantly the expression of proteins involved in several essential physiological and pathophysiological pathways involved in cell–cell interaction and adhesion.

Firstly, we found that BPA promotes downregulation in the expression of podocytes cytoskeleton proteins such as tubulin, vimentin, and podocin. In this regard, George et al.^47^ have shown that tubulin is a direct target of BPA in embryonic and somatic cells where BPA promotes disruption of the microtubule organization. Moreover, vimentin filaments that make up a substantial portion of the cytoskeleton of podocytes cell body and primary and secondary foot processes; it is also likely to be important for cell adhesion and spreading through interactions with actin and filamin trafficking^48^. Furthermore, podocin is a membrane protein that interacts physically and functionally linking the cell membrane to the cytoskeleton and directing its reorganization^49,50^. Downregulation of podocin has been associated with both proteinuria and podocyte survival. These data are in agreement with our previous observation in rodents receiving BPA^43^.

Secondly, BPA also induced a significant decreased in the cytoskeleton-binding and stabilization proteins cofilin-1 and vinculin. Cofilin-1 is an essential regulator for actin filaments recycling that is required for the dynamic nature of podocyte foot processes^51^. Interestingly, higher H_2_O_2_ concentration, an event related to BPA^52^, induces oxidation of cofilin cysteines (C139, C147), resulting in an inhibition of cofilin activity^53,54^, an effect that could lead to proteinuria^55^.

Vinculin, a cytoplasmic protein, couples actin filaments to integrin-mediated cell–matrix adhesions and cadherin-based intercellular junctions^56–58^. Vinculin is an adapter protein that localizes at cell–matrix adhesions and cell–cell junctions required to maintain glomerular barrier integrity^58^. Vinculin has a well-characterized function of stabilizing cell–matrix adhesions by orchestrating the recruitment and release of other cell–matrix adhesion proteins, thereby controlling the strength of adhesion binding to the extracellular matrix. Besides being more susceptible to glomerular injury, mice lacking podocyte vinculin develop altered adhesion and signaling, including albuminuria. Finally, vinculin levels are reported to be altered in human glomerular diseases^58^.

Thirdly, BPA induced the downregulation of essential proteins related to cell adhesion such as E-cadherin, nephrin, and VCAM-1. E-cadherin is a calcium-dependent cell–cell adhesion molecule responsible for the primary cell adhesion system in epithelium^58^. This multiprotein complex interacts with the actin cytoskeleton and physically links cells to each other^59^. This is in accord with our previous findings showing that BPA was able to downregulate nephrin and podocin protein expression in mouse podocytes in culture^43^. VCAM-1 is another protein involved in cell adhesion^60^ that we found to be downregulated by BPA.

Fourth, we found that BPA induced the upregulation of two proteins known to be associated with loss of cell adhesion such as tenascin-C and β-catenin. Tenascin-C is an extracellular matrix glycoprotein that modulates adhesion of cells to fibronectin and thus can be classified as an anti-adhesive, adhesion-modulating extracellular matrix protein^61^. β-catenin is the critical component in the highly conserved canonical Wnt pathway, regulating cell–cell adhesion^62^ as well as serving as a transcription co-factor^63^. Widely expressed, β-catenin is also found in podocytes and plays a pivotal role in cell adhesion and differentiation. Previous studies showed that β-catenin activity is explicitly upregulated in glomerular podocytes in various proteinuric kidney diseases^64,65^. Herein our results clearly indicated that BPA significantly increased the protein expression of tenascin-C as well as β-catenin. An interaction between β-catenin and tenascin-C^66,67^ is known to occur and may also trigger podocytopathy as described in other cell types^68,69^.

As previously mentioned, the proteomic analysis revealed an upregulation of the enzymes superoxide dismutase, glutathione synthetase, and NADPH adrenodoxin oxidoreductase, suggesting that BPA could also trigger the production of damaging oxygen-free radicals. In previous work, we demonstrated that in the endothelium BPA could activate vasoregulatory genes such as angiotensin II and calcium-calmodulin kinase II responsible for endothelial dysfunction and hypertension, through a mechanism involving the uncoupling of endothelial nitric oxide synthase promoting a decreased of NO production and overproduction of oxygen free radicals^42^. Interestingly we found that BPA also promotes a decrease in NO production and an overproduction of superoxide on human podocytes.

Since Tamoxifen was able to abrogate BPA´s effect on podocyte adhesion, we analyzed if this protective effect was associated with the alteration of the expression of adhesion and cytoskeleton proteins promoted by BPA. Herein we observed that Tamoxifen was capable to prevent the alteration of fundamental structural proteins such as VCAM-1, vinculin, E-cadherin, nephrin and podocin on human podocytes.

In conclusion, our data show that BPA promoted a novel type of podocytopathy characterizes by an impairment of podocyte adhesion by altering the expression of adhesion and cytoskeleton proteins. Moreover, BPA diminished podocyte NO production and induced the overproduction of oxygen-free metabolites. Although further translational studies are needed to clarify BPA´s potential role in the pathogenesis and the progression of renal diseases, these data provide a mechanism by which BPA could promote renal damage.

## Methods

### Human podocyte culture

Conditionally immortalized human podocytes (a generous gift from Dr. M Saleem, University of Bristol and Dr. J Nornan, Royal Free Hospital, London, UK) were cultivated as previously described by Saleem et al.^70^. In brief, cells were grown in standard RPMI 1640 medium containing 10% FBS and supplements at the permissive temperature of 33 °C (in 5% CO_2_) to promote cell propagation to 50–80% confluence. After that, cells were shifted to the non-permissive temperature of 37 °C (in 5% CO_2_) to allow terminal differentiation for 15 days. Next, cells were treated with different concentrations of BPA (Aldrich chemistry) and BPA-glucuronide (G-BPA, a conjugated form by the liver) for 1, 2, or 3 days. In all cases, BPA and G-BPA were dissolved in ethanol and then added to the culture medium.

We used a BPA concentration of 10 and 100 nM, within the range that many authors consider as low dose^71,72^ and within the concentration range to which the human being is exposed. It has been described that the general population has a concentration of BPA that ranges between 1 and 10 nM, while workers with high exposure to BPA can reach a concentration of 100 nM^73–75^. Furthermore, it has been described that surgical interventions or hemodialysis in the hospital environment can increase the exposure to BPA even more^44–46^.

We also use 100 nM Tamoxifen (H6278 Sigma-Aldrich) and 100 nM ICI 182,780^76–78^ (dissolved in ethanol) as ER blockers 1 h before adding the BPA treatment.

### MTT cell viability assay

After BPA treatment (3 days), 50 µl of MTT (5 mg/ml) were added to each well in 500 µl of the medium, and the plates were incubated for 1 h 30 min at 37 °C. Then, DMSO (Sigma Aldrich) was added to solubilize the formazan crystals. The absorbance was measured at a test wavelength of 570 nm with a reference wavelength of 690 nm^43^.

### Adhesion assay

In order to perform an adhesion cell assay that would allow quantifying the number of cells able to reattach to the culture dish after 48 h of BPA exposition, Human Podocytes were treated with 1, 10 and 100 nM BPA, 100, 1000 nM Glucuronide-BPA and 100 nM Tamoxifen and ICI or the respective control for 48 h. The medium was removed, and cells were exposed to Accutase (Thermo Fisher Invitrogen) until all cells were suspended, which was optically controlled. Hereafter, 25,000 cells were left to settle again in standard RPMI 1640 medium containing 10% FBS for 60 min. Then the medium was removed, the P24 was washed with PBS, and attached cells were measured determining MTT cell viability or count by simple staining with violet crystal^79^.

### Transcriptomic and proteomic studies on human podocytes

For both transcriptomic and proteomic analyses human podocytes incubated 48 h with 100 nM BPA.

### Microarray analysis

#### Library construction protocol

1 µg of total RNA samples, containing ERCC ExFold RNA Spike-In Mixes (Ambion 4456739), was used. The average sample RNA Integrity Number was 9.8 when assayed on an Agilent 2100 Bioanalyzer. PolyA+ fraction was purified and randomly fragmented, converted to double-stranded cDNA and processed through subsequent enzymatic treatments of end-repair, dA-tailing. This was followed by the ligation to adapters as in Illumina's "TruSeq Stranded mRNA Sample Preparation Part # 15031047 Rev. D" kit (this kit incorporates dUTP during 2nd strand cDNA synthesis, which implies that only the cDNA strand generated during 1st strand synthesis is eventually sequenced).

PCR completed the Adapter-ligated library with Illumina PE primers (10 cycles). The resulting purified cDNA library was applied to an Illumina flow cell for cluster generation and sequenced on an Illumina instrument (Illumina HiSeq2500) by following the manufacturer's protocols^80^.

For primary data processing, image analysis, per-cycle base calling, and quality score assignment was performed with Illumina Real-Time Analysis software. Conversion of Illumina BCL files to bam format was performed with the Illumina2bam tool (Wellcome Trust Sanger Institute—NPG).

### Proteomic analysis: iTRAQ assay

#### Protein digestion and tagging with TMT simplex reagent

For digestion, 40 µg of protein from each condition were precipitated by methanol-chloroform method. Protein pellets were resuspended and denatured, as previously described by Méndez et al.^81^. The resulting peptides were subsequently labeled using TMT-six-plex Isobaric Mass Tagging Kit (Thermo Scientific, Rockford, IL, USA) according to the manufacturer's instructions as follows: 126: C-1; 127: BFA-1; 128: C-2; 129: BFA-2; 130: C-3; 131: BFA-3. Three biological replicates of each condition were analyzed in this study. After labeling, the samples were pooled, evaporated to dryness and stored at − 20 °C until the LC–MS analysis.

#### Nano-liquid chromatography and mass spectrometry analysis

A 1 µg aliquot of the labeled mixture was subjected to 1D-nano LC ESI-MSMS analysis using a nano-liquid chromatography system (Eksigent Technologies nanoLC Ultra 1D plus, SCIEX, Foster City, CA) coupled to high-speed Triple TOF 5600 mass spectrometer (SCIEX, Foster City, CA) with a Nanospray III source. The analytical column used was a silica-based reversed-phase Acquity UPLC M-Class Peptide BEH C18 Column, 75 µm × 150 mm, 1.7 µm particle size, and 130 Å pore size (Waters). The trapping column was a C18 Acclaim PepMap 100 (Thermo Scientific), 100 µm × 2 cm, 5 µm particle diameter, 100 Å pore size, switched on-line with the analytical column. The loading pump delivered a solution of 0.1% formic acid in water at 2 µl/min. The nano-pump provided a flow-rate of 250 nl/min and was operated under gradient elution conditions. Peptides were separated using a 250 min gradient ranging from 2 to 90% mobile phase B (mobile phase A: 2% acetonitrile, 0.1% formic acid; mobile phase B: 100% acetonitrile, 0.1% formic acid). The injection volume was 5 µl.

Data acquisition was performed with a TripleTOF 5600 System (SCIEX, Foster City, CA). Data were acquired as previously described by Méndez et al.^81^.

### Data analysis

MS/MS spectra were exported to mgf format using Peak View v1.2.0.3 following by proteomic search engines (Mascot Server 2.5.1, OMSSA 2.1.9, X!TANDEM 2013.02.01.1, and Myrimatch 2.2.140) against a composite target/decoy database built from the 71,785 Homo sapiens sequences at UniProt (proteome ID UP000005640, January 2018) plus some commonly occurring contaminants. Correctly identified peptides from an initial X!TANDEM search with a mass error tolerance of 35 ppm was used to recalibrate parent ion mass measurements in all spectra using linear models. All search engines were then configured as previously described by Méndez et al.^81^. All analyses were conducted using software from Proteobotics (Madrid, Spain).

### Western blot

After electrophoresis of total cell proteins, samples were immunoblotted as previously reported^82,83^. Membranes were then incubated overnight at 4 °C with the following polyclonal antibodies [dilution, -fold]: anti-VCAM1 antibody (Abcam,) [5000], anti-E-Cadherin antibody (BS Transduction Laboratories) [1000], anti-tenascin-C antibody (Santa Cruz Biotechnology, Santa Cruz CA) [500], anti- NPHS2 antibody (Abcam) [5000], anti-nephrin antibody (Abcam) [5000] and anti-tubulin (Sigma–Aldrich, Saint Louis, MO [5000]. Anti-vimentin (Santa Cruz Biotechnology) [1000], anti-cofilin-1 (Santa Cruz Biotechnology) [1000], anti-vinculin (Santa Cruz Biotechnology) [1000], anti-ILK (Santa Cruz Biotechnology) [1000], anti-β-catenin (Santa Cruz Biotechnology) [2000].

Coomassie staining (Sigma) of the membrane was used as an internal loading control. Blots were analyzed by densitometric scanning with Image J. Western blot studies in cultured cells were performed in at least three independent experiments, and a representative figure is shown.

### Immunocytochemistry

Cells were fixed with 4% paraformaldehyde for 10 min and rinsed in phosphate buffer saline (PBS). Cells were then incubated for 30 min with 5% normal donkey serum in PBS to block nonspecific binding. Afterward, cells were incubated overnight at 4 °C with primary antibodies [1:200] and then washed with PBS. Finally, cells were incubated with α-rabbit-Alexa-Fluor 488 or α-mouse-Alexa-Fluor 488, both diluted 1:1500 for 1 h in the darkness. Slides were then washed and mounted with ProLong Gold antifade reagent with DAPI (Invitrogen). Detection was performed by confocal laser scan microscopy LEICA TCS-SL (Heidelberg, Germany). In some cases, protein detection was performed with DAB chromogen; then, after primary antibodies incubation, the cells were washed and incubated in primary antibodies amplifier Quanto (Ultravision Quanto detection system–peroxidase, Master Diagnóstica, Granada, Spain) for 10 min. After an extensive wash in PBS, the cells were incubated in polymer Quanto for 10 min. The peroxidase activity was detected using the DAB kit (Master Diagnóstica). Cover slides were counterstained with hematoxylin, dehydrated, cleared in xylene, and mounted in Entellan.

### Nitric oxide production by human podocytes

Nitric oxide production was measured in podocytes loaded with diaminofluorescein diacetate (DAF-DA; 2 µM), and propidium iodide (1 µg/sample) was used to determine cell viability as previously described (58). After exposure to different experimental conditions; PBS (control) and BPA (100 nM) for 24 h, cells were and labeled with the fluorochrome at 37 °C for 1 h 30 min, trypsin dispersed and followed by cytofluorometric analysis with a fluorescence-activated cell sorter (FACS) scanner (Becton Dickinson, New York, NY, USA). Ten thousand events were analyzed for each condition.

### Superoxide anion production

Superoxide reacts with dihydroethidium (DHE), forming 2-hydroxyethidium. Podocytes were incubated with PBS (control) and BPA (100 nM) for 24 h and treated with 10 µM apocynin for 1 h to inhibit NADPH oxidase. After exposure to different experimental conditions, cells were trypsin dispersed and labeled with the fluorochrome (DHE) for 30 min, followed by cytofluorometric analysis with a FACS scanner. A total of 10,000 events were analyzed for each condition^42^.

### Statistical analysis

Data were reported as the mean ± SEM. The Kolmogorov–Smirnov test assessed normal distribution. To determine the effects of BPA, one-way ANOVA or Kruskal–Wallis followed by a Bonferroni or Dunn´s test, respectively, was carried out. The *p* values presented in figures and tables corresponded to post hoc test. All statistical analyses were performed using the GraphPad Prism 7.0 software (GraphPad Software Inc., San Diego, CA, USA). Differences were considered statistically significant at *p* < 0.05. In the case of the proteomics test, the corresponding proteins data were analyzed using the *q* value < 0.05^84,85^.

## Supplementary information

Supplementary figures
