# Supplementary material for: Bisphenol A impaired cell adhesion by altering the expression of adhesion and cytoskeleton proteins on human podocytes
Source: Sci Rep. 2020 Oct 6;10:16638. doi: 10.1038/s41598-020-73636-6 (PMC7538920; doi:10.1038/s41598-020-73636-6)

Supplementary material

“Bisphenol A impaired cell adhesion by altering the expression of adhesion and cytoskeleton proteins on human podocytes“

Rafael Moreno-Gómez-Toledano
María I. Arenas
Clara González-Martínez
Nuria Olea-Herrero
Paula Reventún
Michele Di Nunzio
Sandra Sánchez-Esteban
Eduardo Arilla-Ferreiro
Marta Saura
Ricardo J. Bosch

Supplementary figure 1.- Vimentin

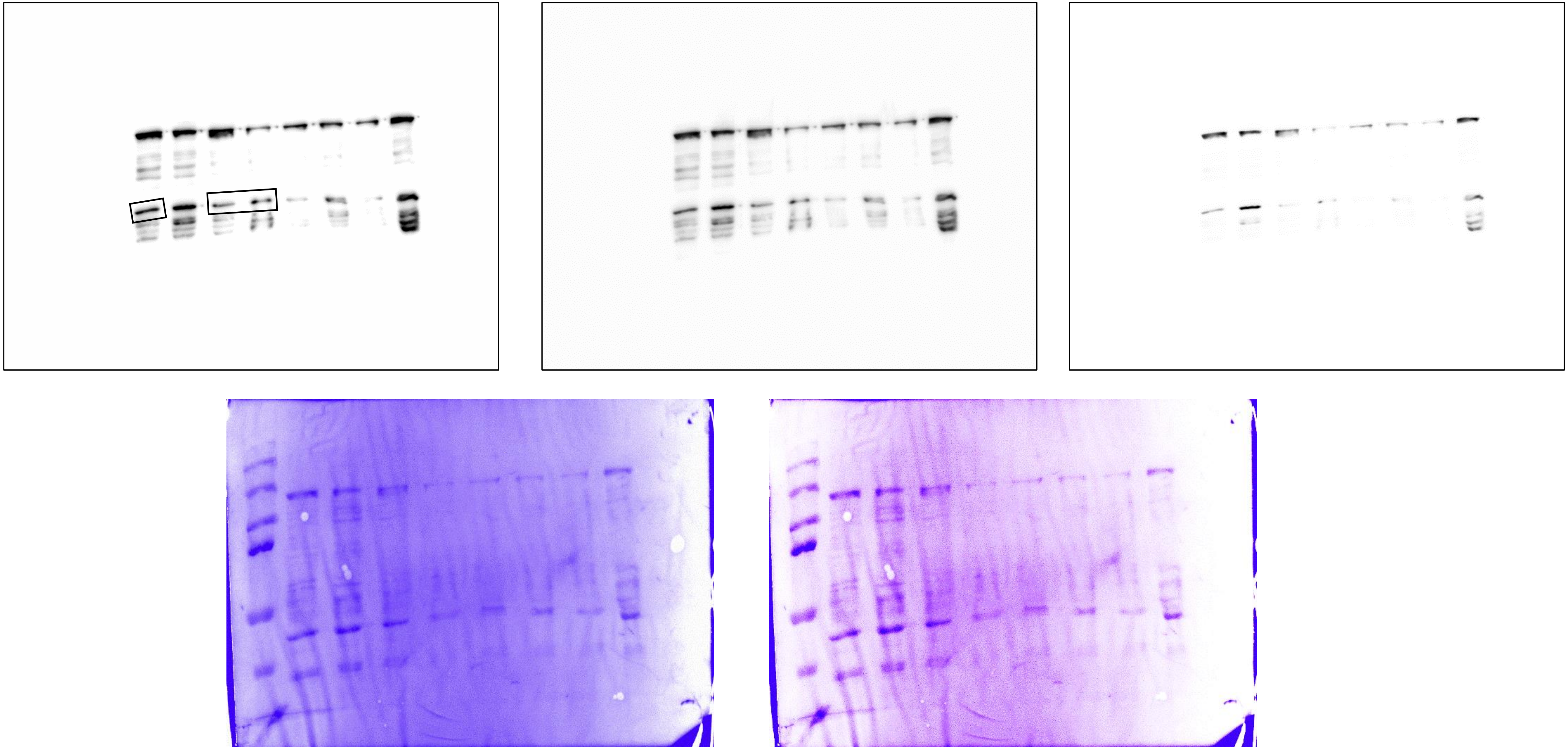

Supplementary figure 2.- Tubulin

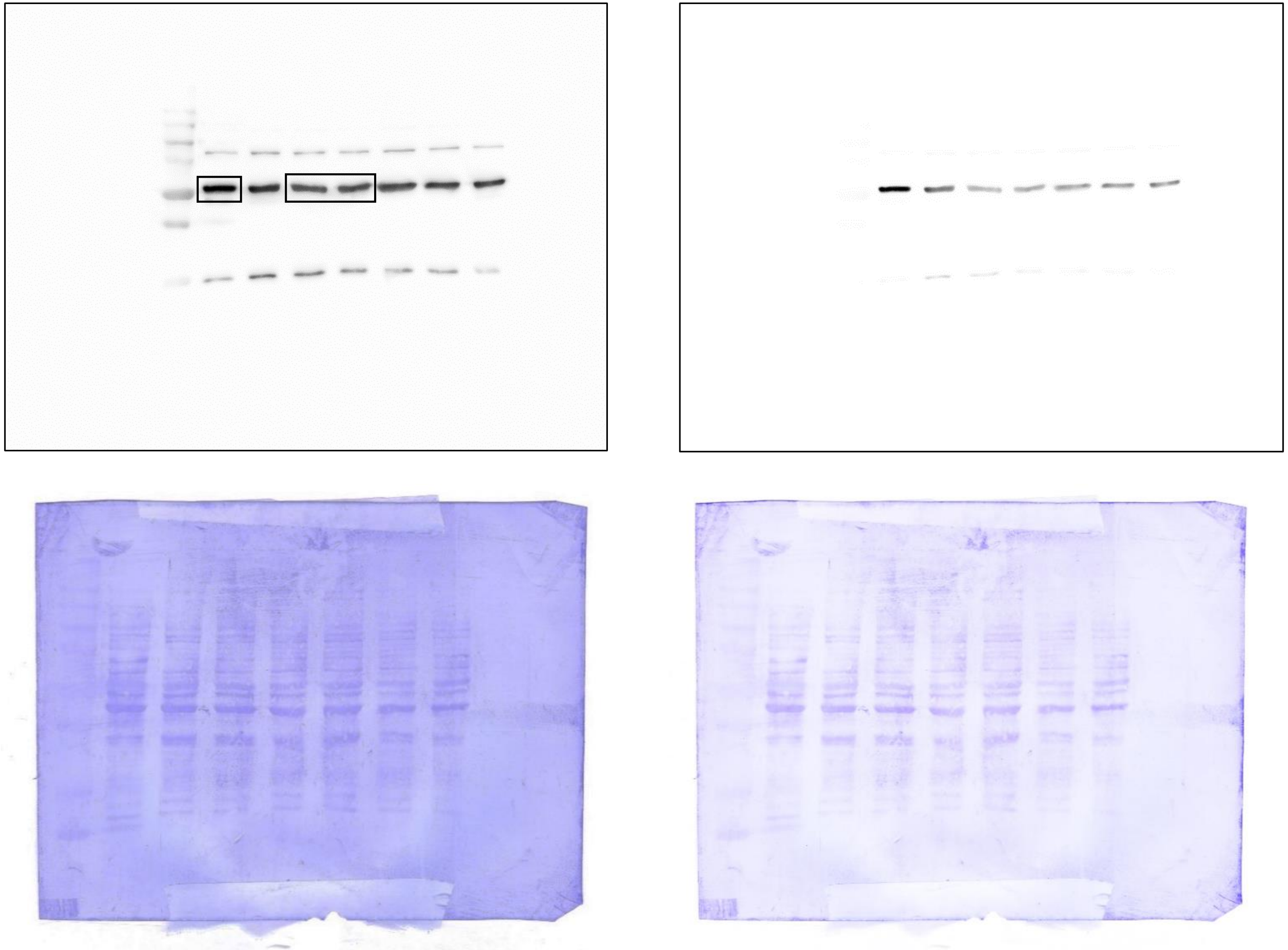

Supplementary figure 3.- Podocin

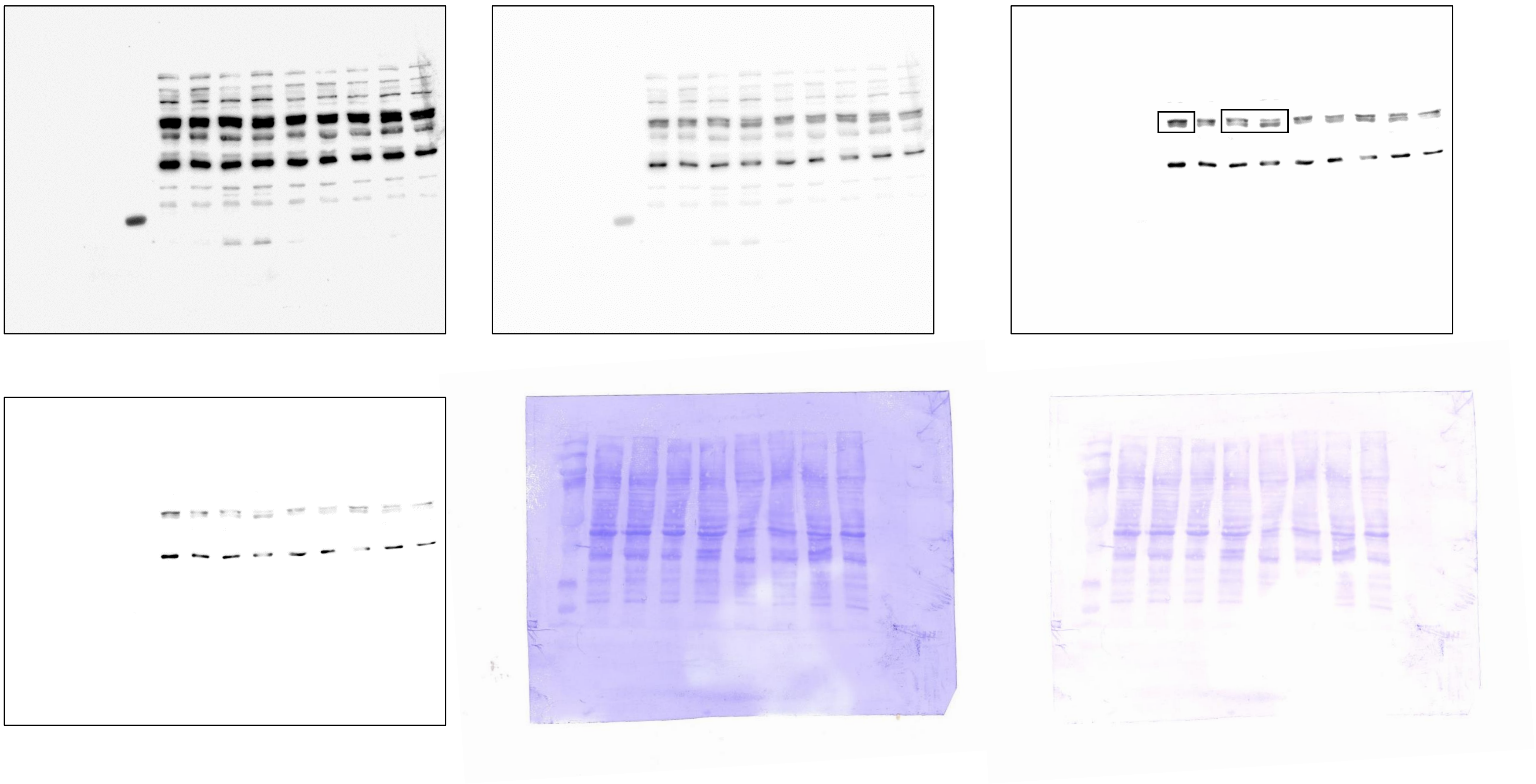

Supplementary figure 4.- Cofilin

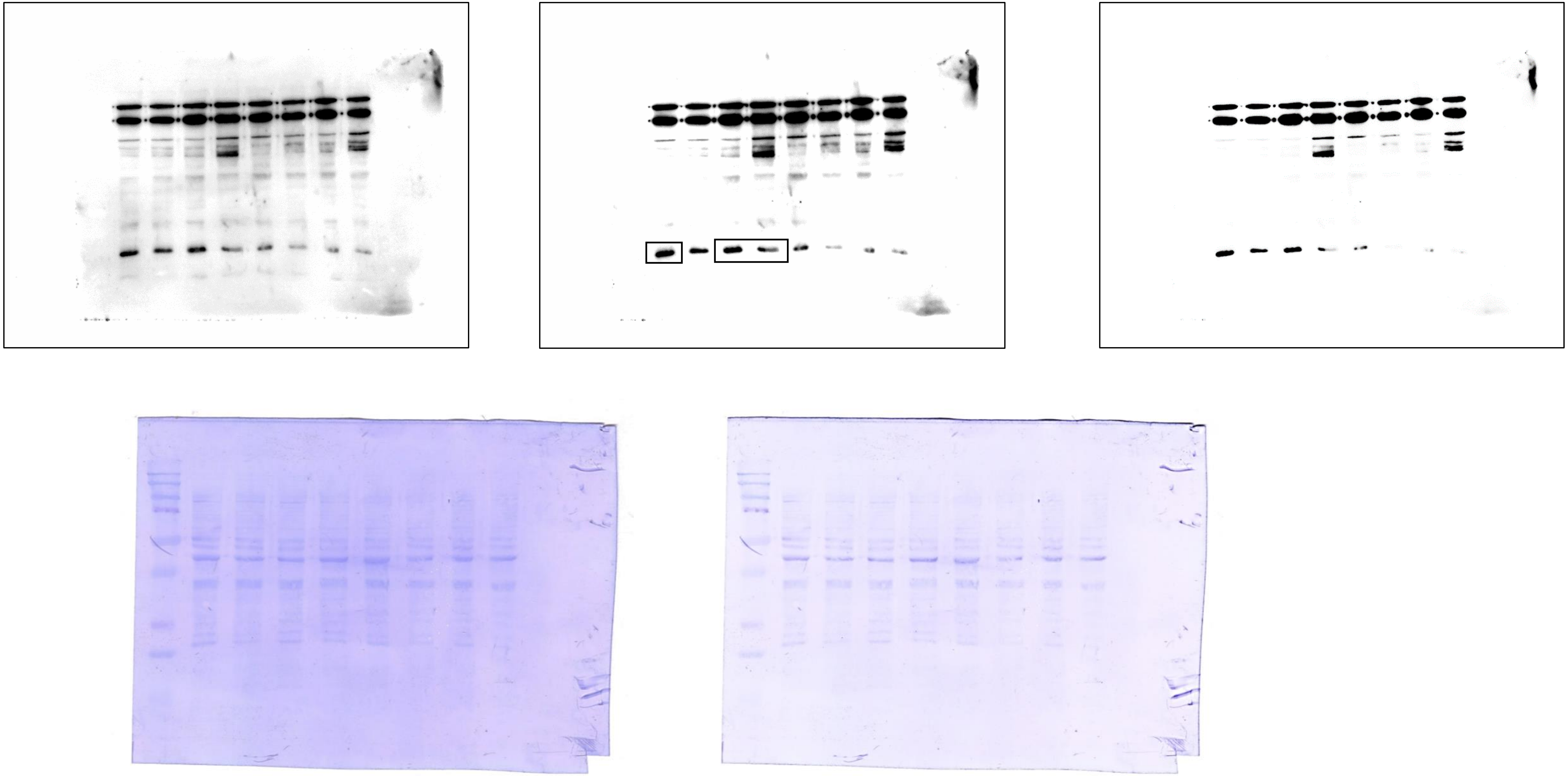

Supplementary figure 5.- Vinculin

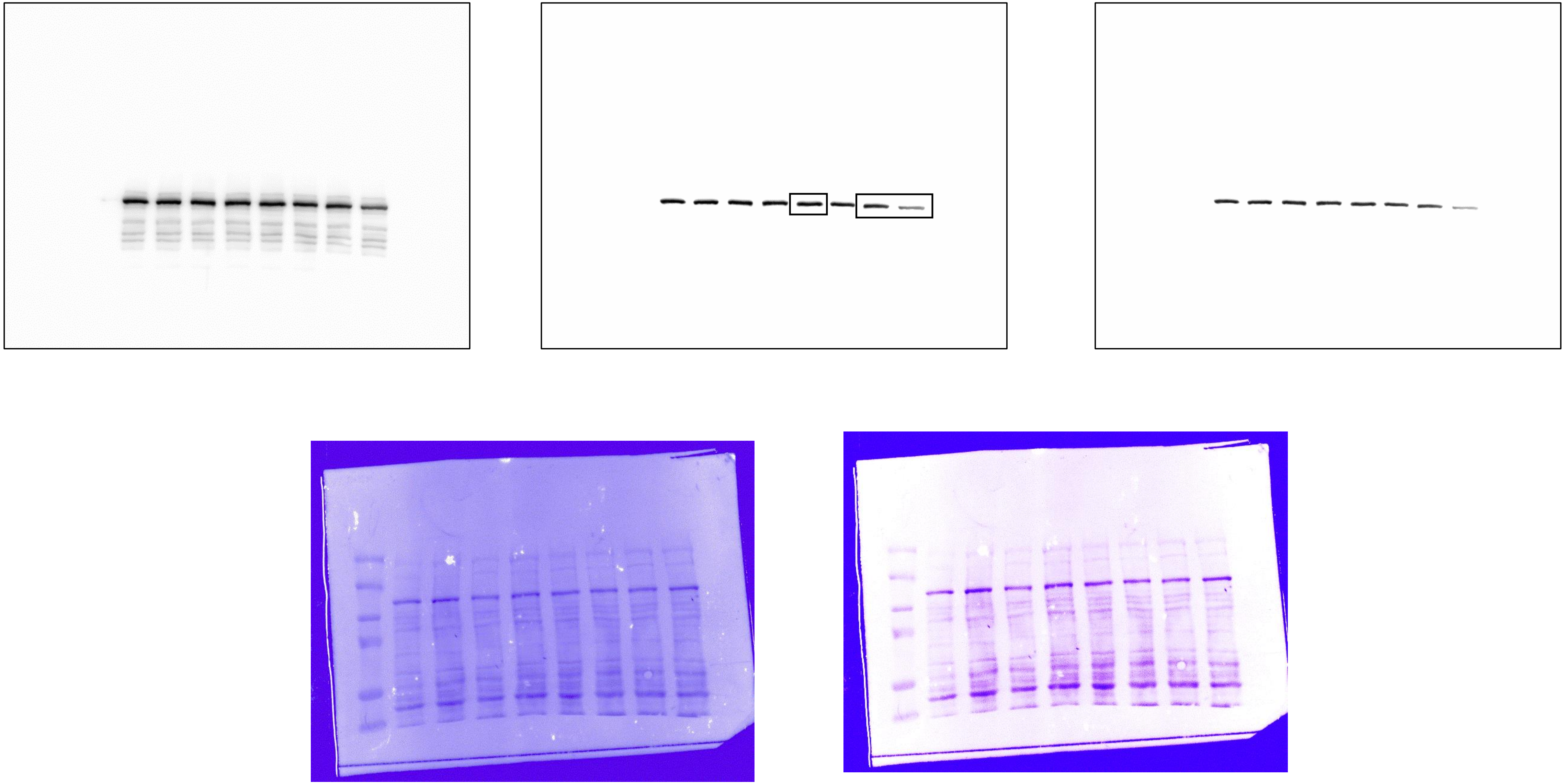

Supplementary figure 6.- E-cadherin

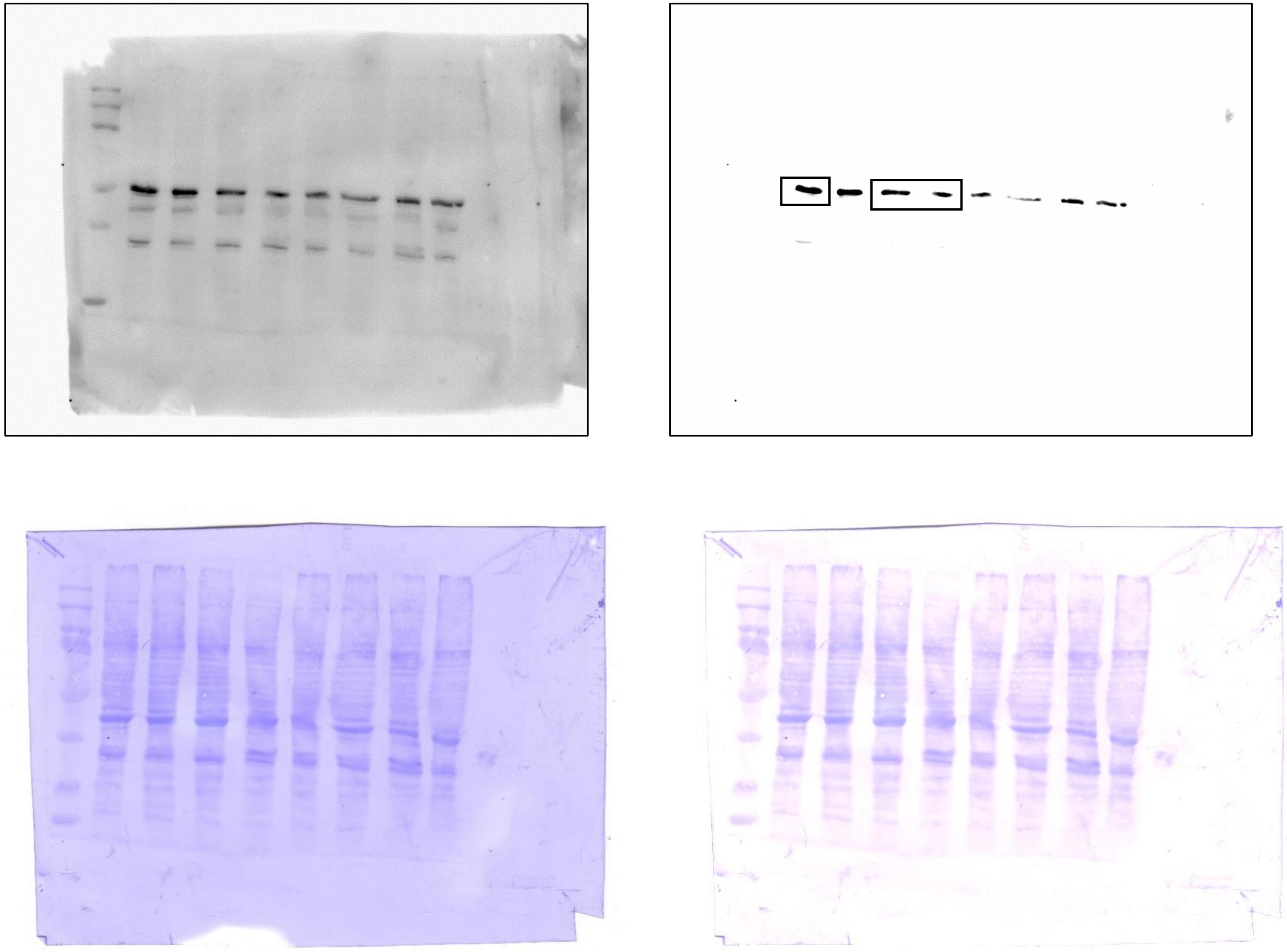

Supplementary figure 7.- Nephrin

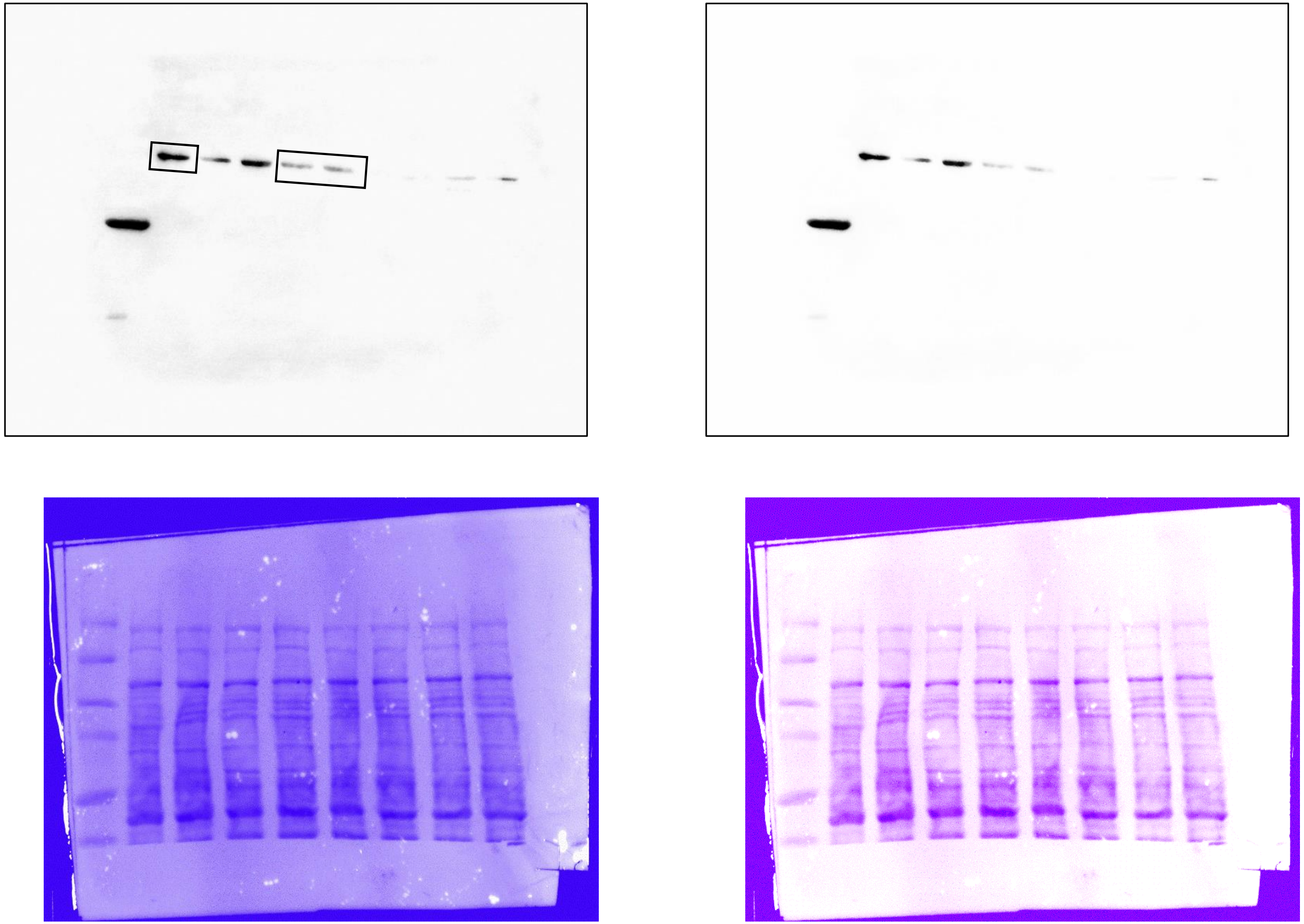

Supplementary figure 8.- V-CAM

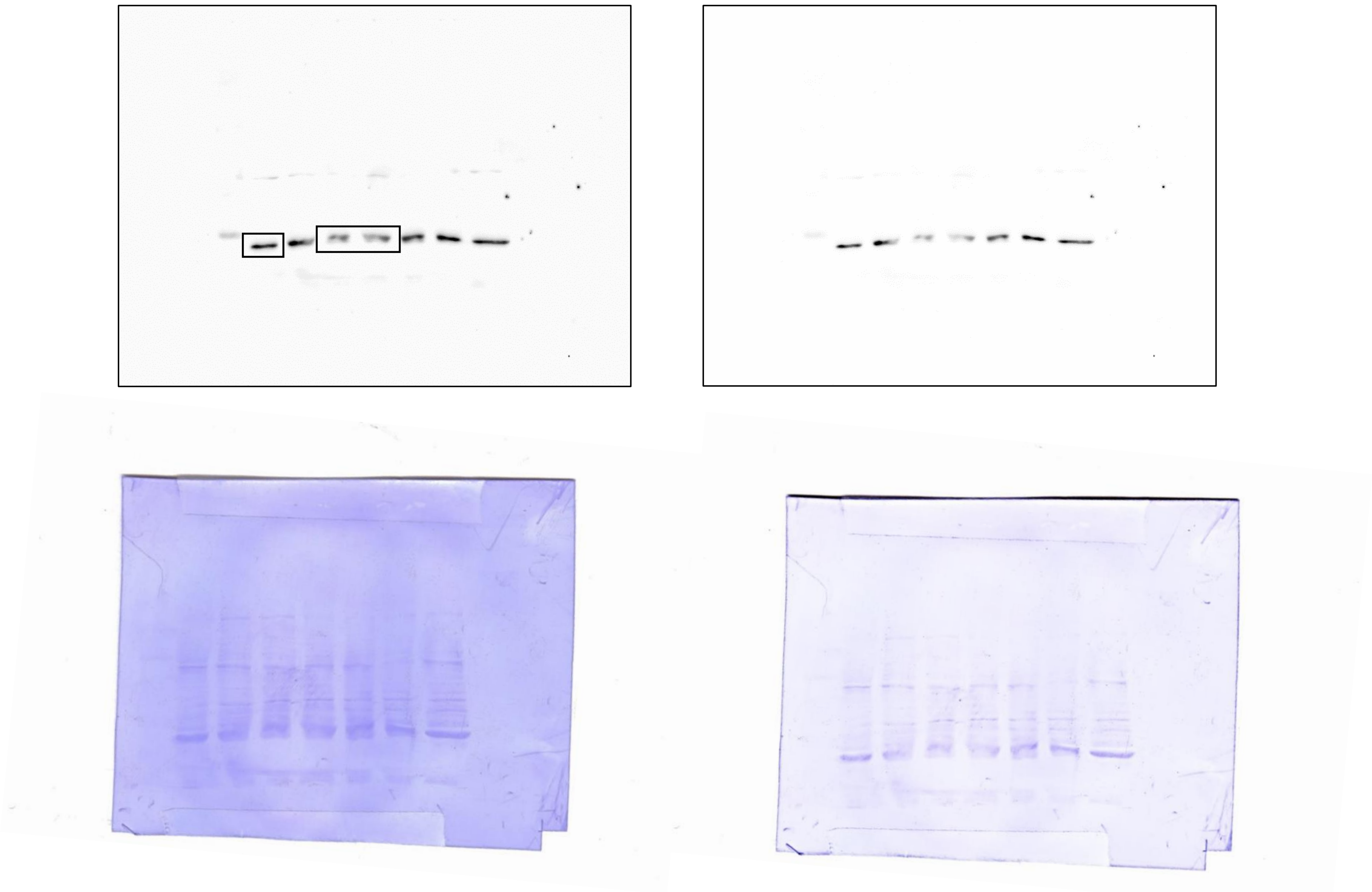

Supplementary figure 9.- Tenascin-C

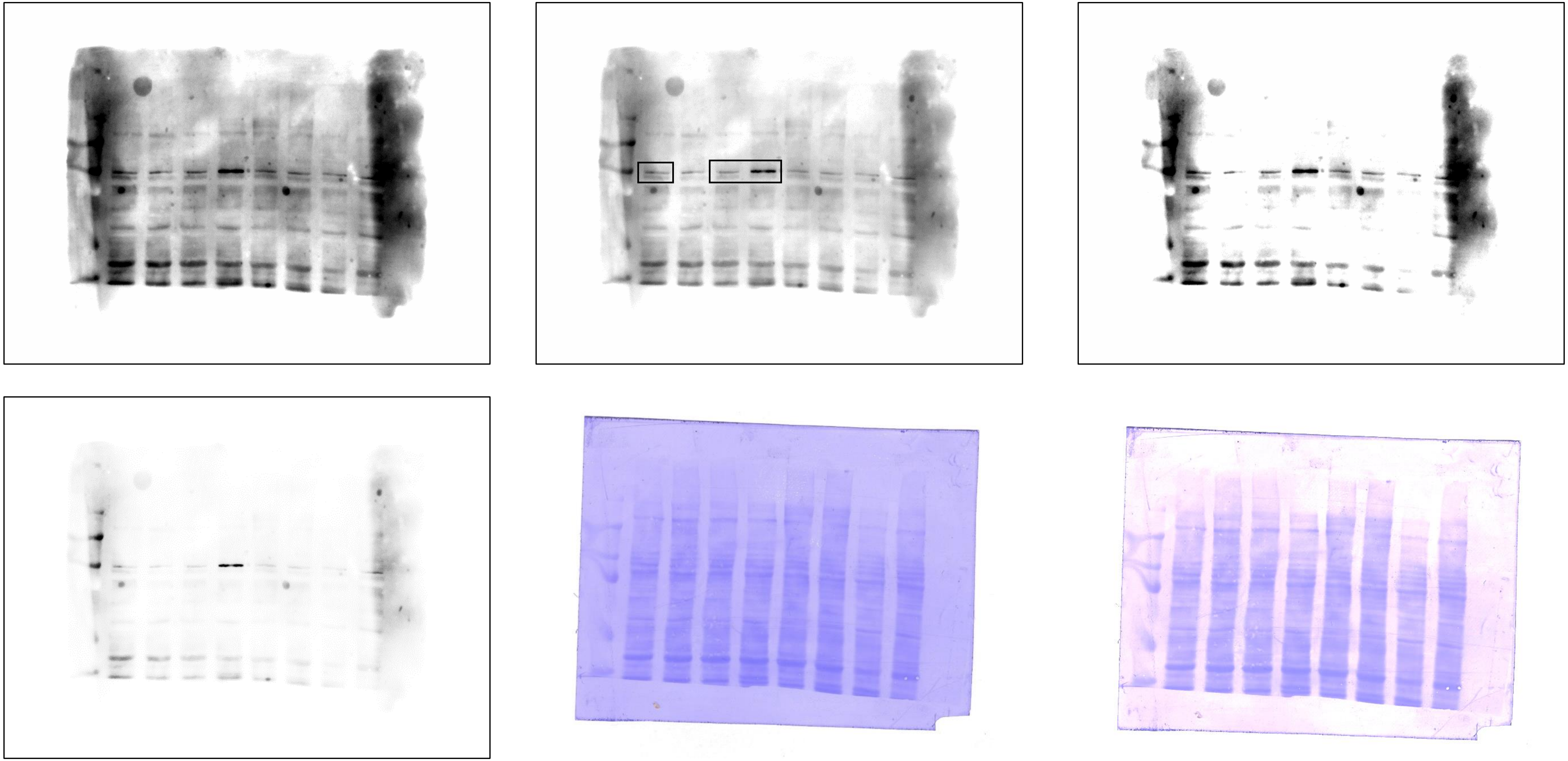

Supplementary figure 10.- β-catenin

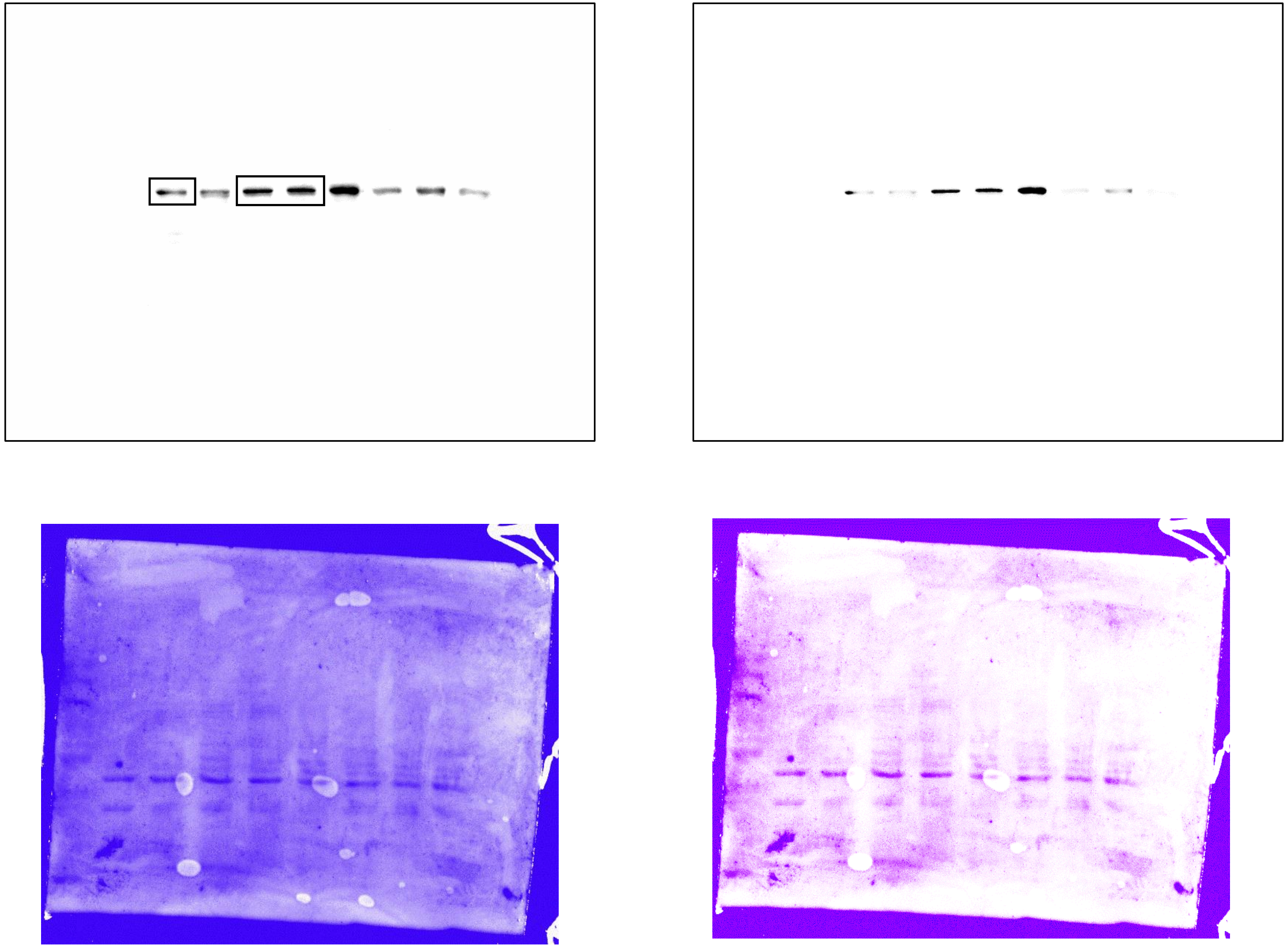

Supplementary figure 11.- V-CAM (Tamoxifen experiments)

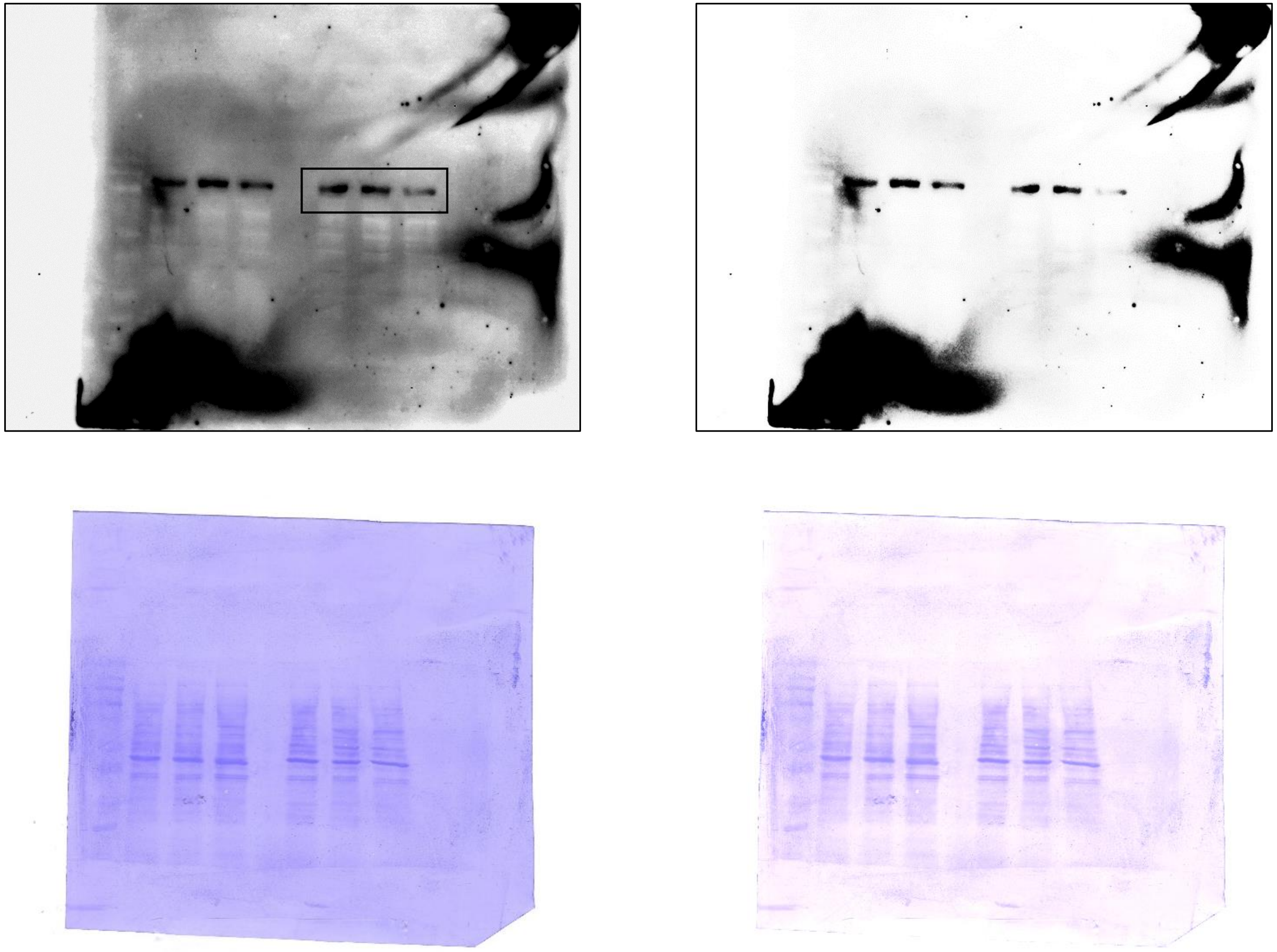

Supplementary figure 12.- Vinculin (Tamoxifen experiments)

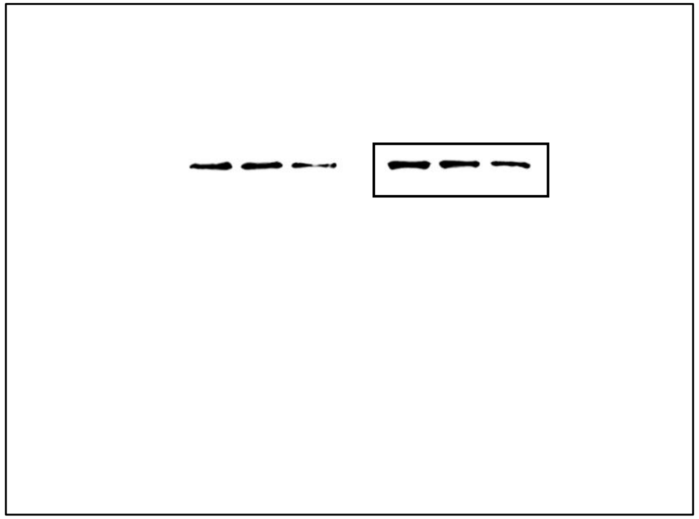

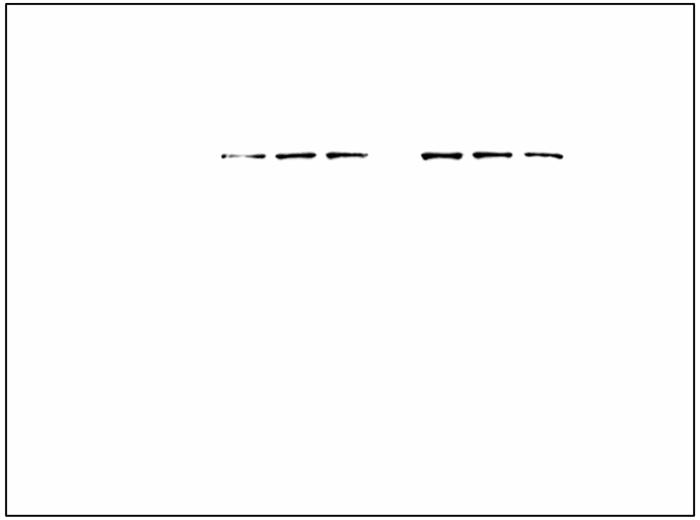

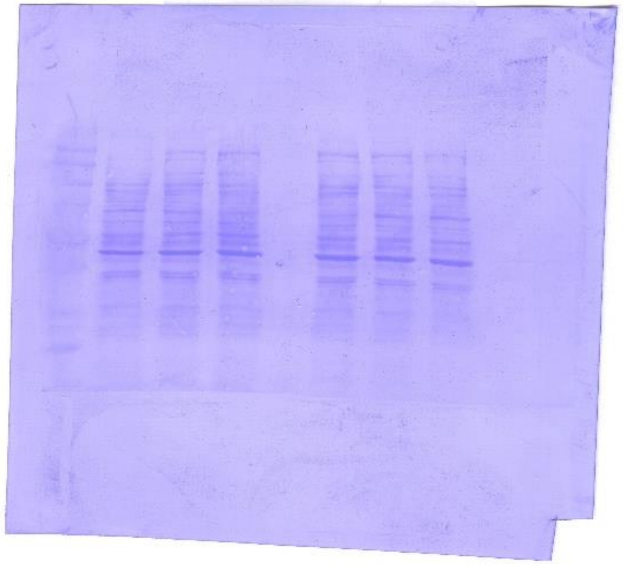

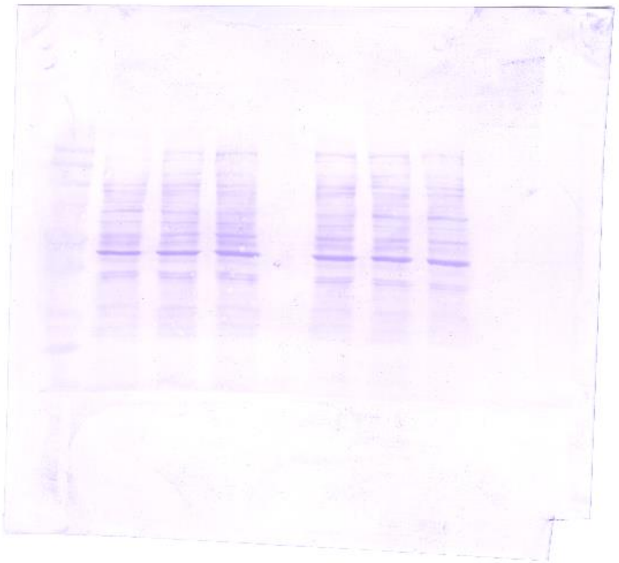

Supplementary figure 13.- E-cadherin (Tamoxifen experiments)

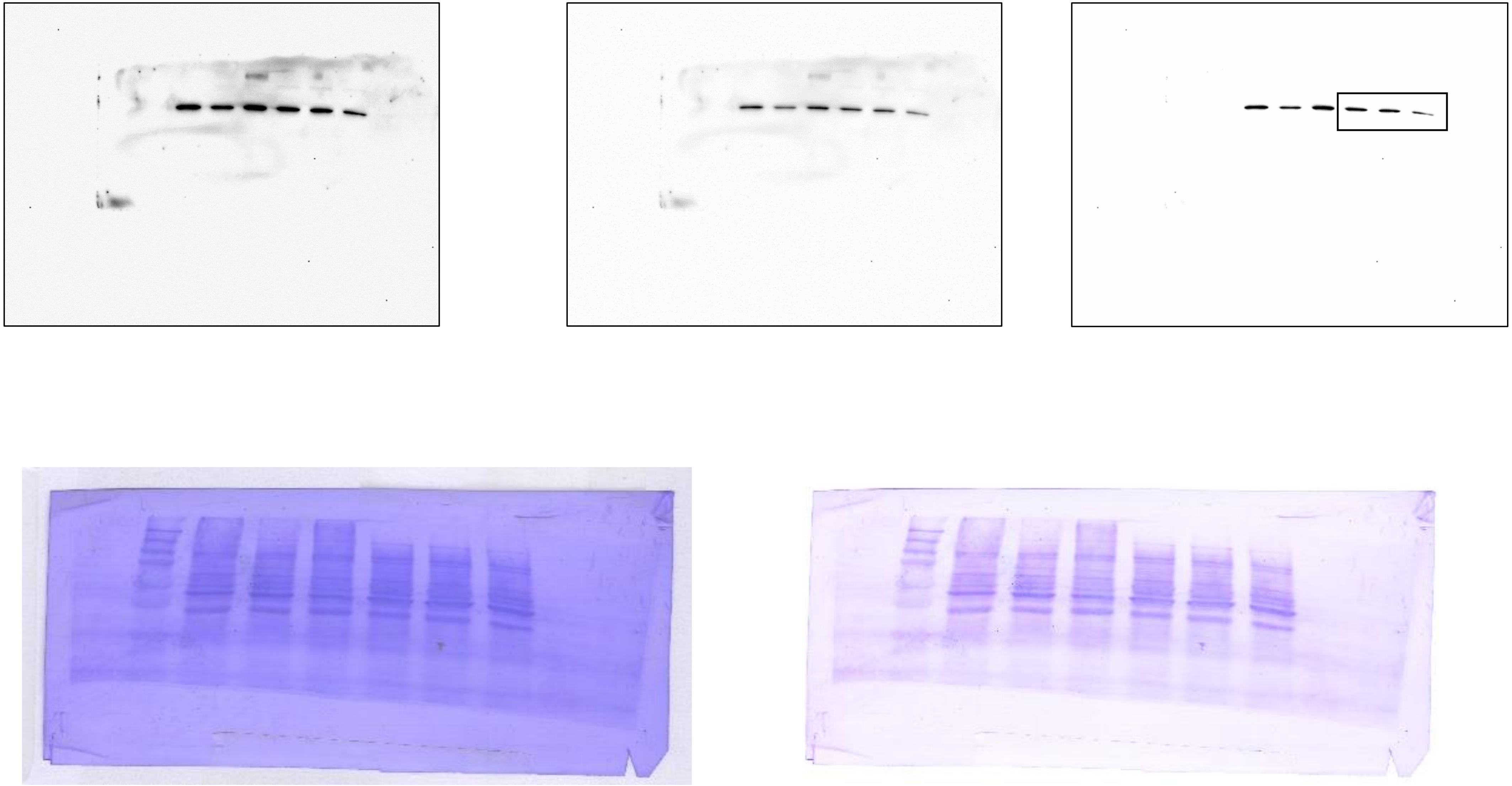

Supplementary figure 14.- Podocin (Tamoxifen experiments)

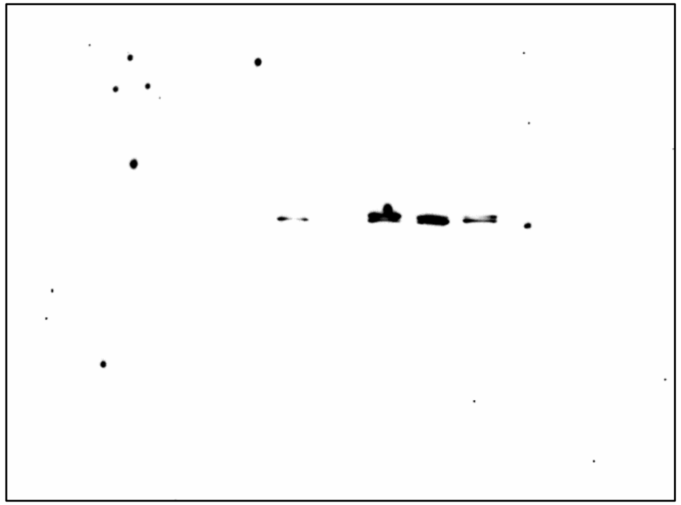

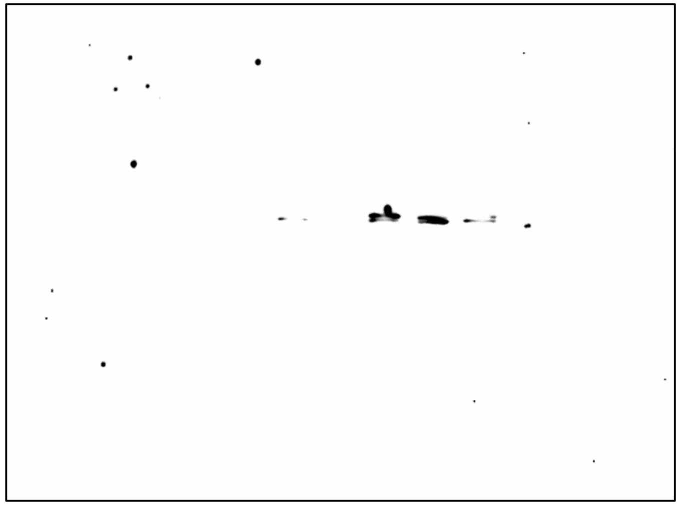

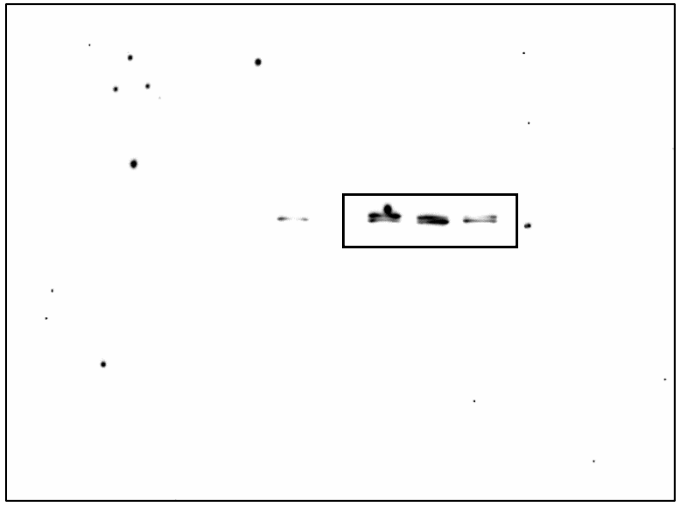

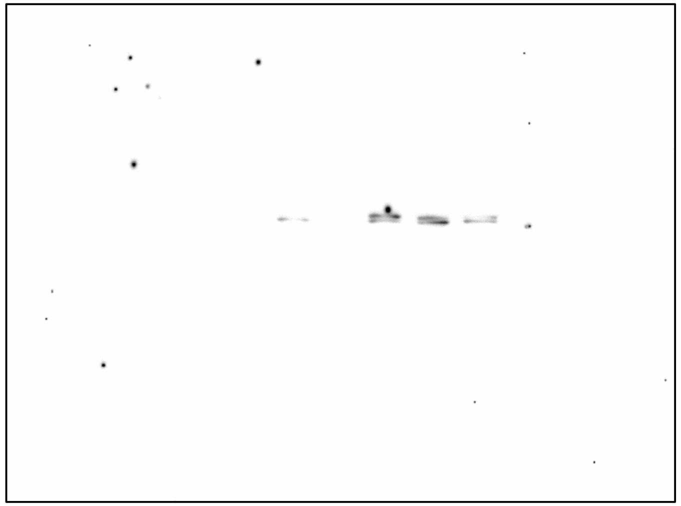

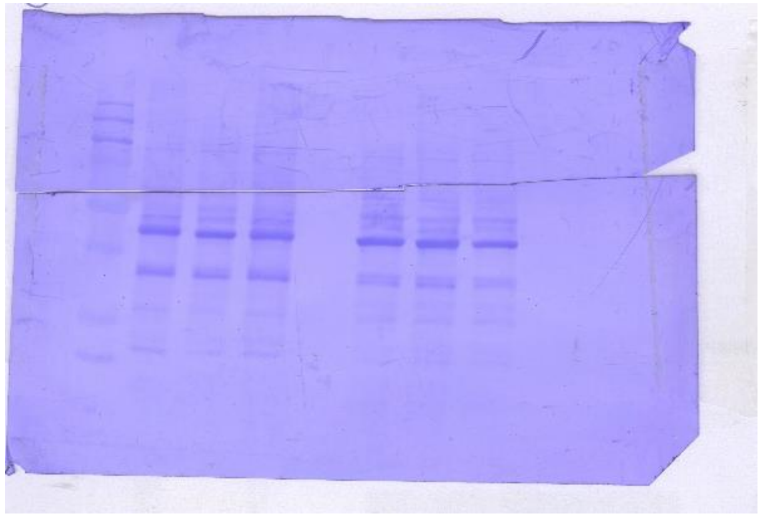

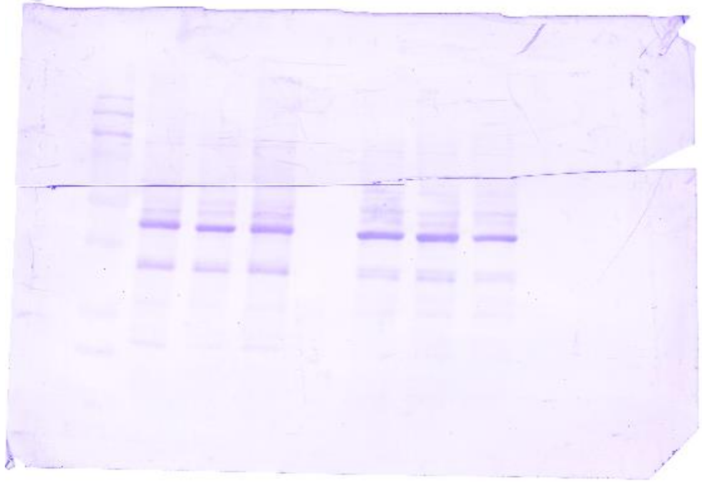

Supplementary figure 15.- Nephrin (Tamoxifen experiments)

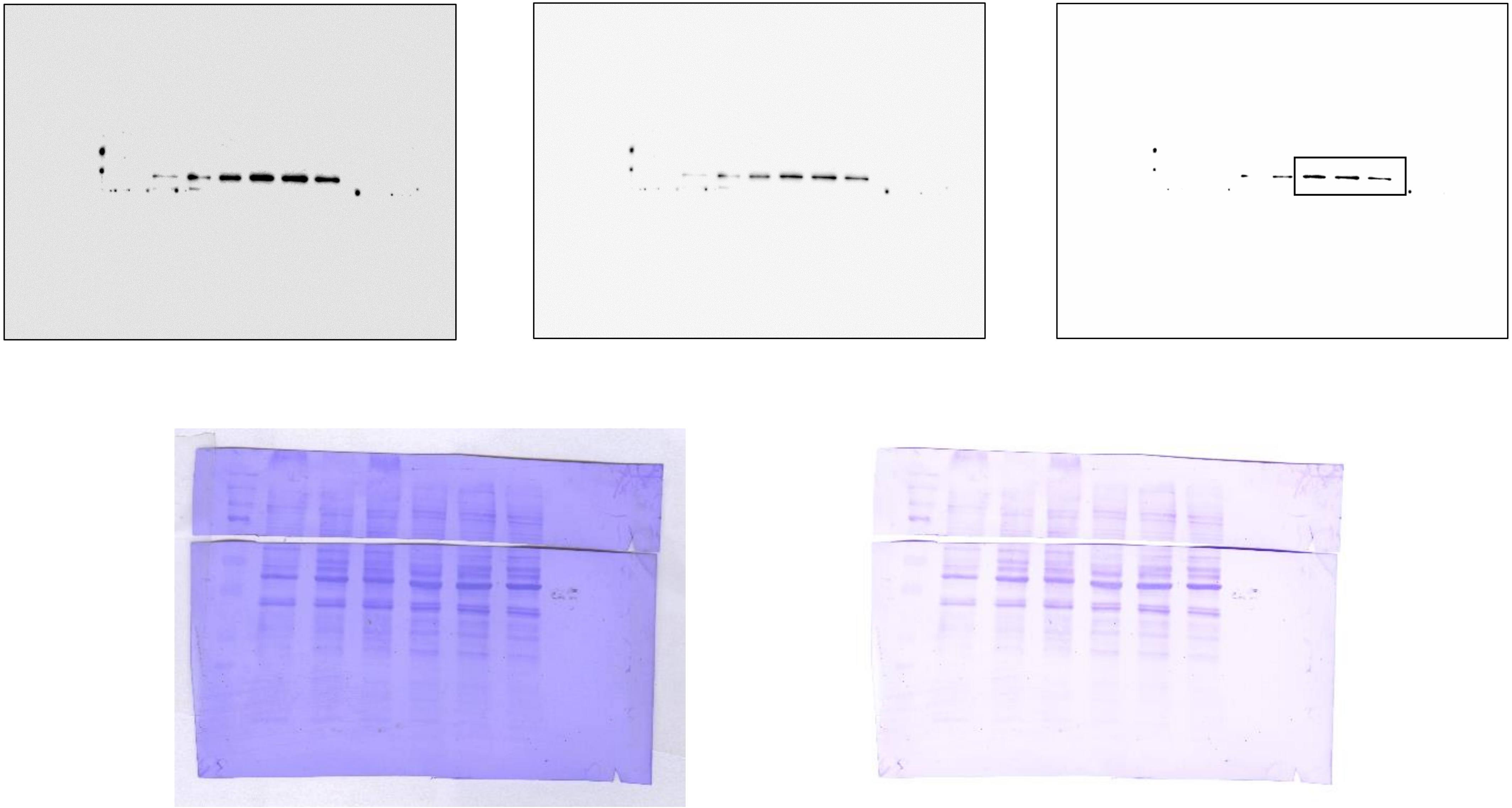

Supplement: Supplementary file 1 — Supplementary figures [file 41598_2020_73636_MOESM1_ESM.pdf]
